# Structural basis of chiral wrap and T-segment capture by *Escherichia coli* DNA gyrase

**DOI:** 10.1073/pnas.2407398121

**Published:** 2024-11-26

**Authors:** Elizabeth Michalczyk, Zuzanna Pakosz-Stępień, Jonathon D. Liston, Olivia Gittins, Marta Pabis, Jonathan G. Heddle, Dmitry Ghilarov

**Affiliations:** ^a^Doctoral School of Exact and Natural Sciences, Jagiellonian University, Kraków 30-348, Poland; ^b^Malopolska Centre of Biotechnology, Jagiellonian University, Kraków 30-387, Poland; ^c^Centre for Programmable Biological Matter, School of Biological and Biomedical Sciences, Durham University, Durham DH1 3LE, United Kingdom; ^d^Department of Molecular Microbiology, John Innes Centre, Norwich NR4 7UH, United Kingdom

**Keywords:** topoisomerase, antibiotics, molecular machine, DNA-binding protein, DNA crossover

## Abstract

Type II topoisomerase DNA gyrase transduces the energy of ATP hydrolysis into the negative supercoiling of DNA. The postulated catalytic mechanism involves stabilization of a chiral DNA loop followed by the passage of the T-segment through the temporarily cleaved G-segment resulting in sign inversion. The molecular basis for this is poorly understood as the chiral loop has never been directly observed. We have obtained high-resolution cryoEM structures of *Escherichia coli* gyrase with chirally wrapped 217 bp DNA with and without the fluoroquinolone moxifloxacin (MFX). Each structure constrains a positively supercoiled figure-of-eight DNA loop stabilized by a GyrA β-pinwheel domain which has the structure of a flat disc. By comparing the catalytic site of the native drug-free and MFX-bound gyrase structures both of which contain a single metal ion, we demonstrate that the enzyme is observed in a native precatalytic state. Our data imply that T-segment trapping is not dependent on the dimerization of the ATPase domains which appears to only be possible after strand passage has taken place.

## Significance

DNA topoisomerases are ubiquitous enzymes in both prokaryotes and eukaryotes which work by selectively trapping DNA crossings of defined chirality. Because of complex organization and high flexibility, structural characterization of the mechanism of these essential enzymes proved difficult. A bacterial topoisomerase DNA gyrase is unique in its ability to negatively supercoil DNA using energy of ATP. In this work, we demonstrate the molecular basis of how gyrase constrains a positively supercoiled loop, and propose an updated mechanism of enzyme catalysis.

Molecular motors consume energy, typically in the form of nucleotide triphosphates, to overcome thermal fluctuations and produce unidirectional motion. A few well-studied examples include kinesin, myosin, and F1-ATPase; however, exactly how localized energy consumption in the form of nucleotide binding and release results in nm-scale directional movements remains a fundamental question (1). Understanding of the organizational principles of molecular machines is important for the manipulation of their activities and design of artificial nature-inspired nanoscale devices.

DNA gyrase is a bacterial type II topoisomerase belonging to the gyrase-Hsp90-kinase-MutL (GHKL) ATPase family: members of this group (DNA topoisomerases, DNA repair proteins, heat shock proteins, and, recently, prokaryotic and eukaryotic immunity proteins) use ATP to trigger dimerization and transition through distinct conformational steps transducing energy into mechanistic outcomes (2–4). Gyrase is essential in bacteria for both removing positively supercoiled DNA in front of the progressing RNA polymerase and introducing negative supercoiling required for chromosomal homeostasis. It directly and indirectly affects virtually all genomic transactions in the cell (5). As such, gyrase is also a successful target for antibiotics, with fluoroquinolones being the most clinically important group (6).

**Fig. 1.**
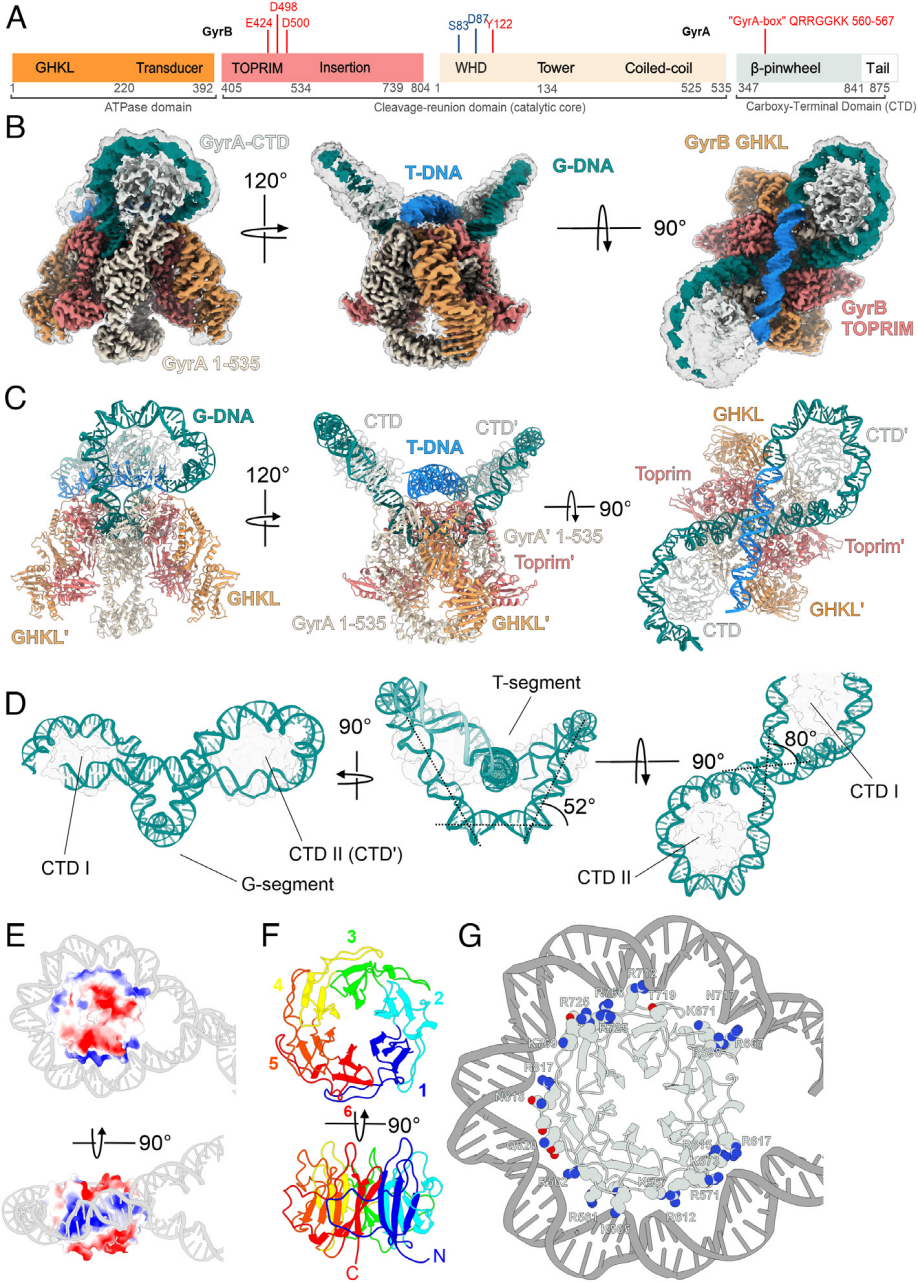
Overall architecture of chirally wrapped *E. coli* gyrase complex and structure of a positively supercoiled DNA loop. (*A*) Domain structure of GyrB (*Left*) and GyrA (*Right*) subunits. Key amino-acid residues important for catalysis and metal binding (red) and fluroquinolone resistance (blue) are indicated. The same color scheme is used throughout the manuscript: GHKL – orange, Toprim – coral, GyrA core region – beige, GyrA CTD & tail – mint white. (*B*) Different views of the consensus cryoEM density map for the **Gyr-Mu217** complex presented at two contour levels (10σ and 5σ). The sharpened 10σ map is colored according to the color scheme above; the G-segment DNA is colored teal and the T-segment DNA light blue. (*C*) Corresponding views of the atomic model of the complete **Gyr-Mu217** complex; protein and DNA are shown in cartoon representation. (*D*) The modeled DNA loop presented in isolation with both CTDs shown as transparent contours. Angles between DNA segments are indicated. (*E*) Surface representation of an isolated CTD colored by Coulombic potential (blue – positive, red – negative, measured by ChimeraX) with the Mu217 right arm wrapped around and shown as a cartoon representation. (*F*) A cartoon representation of a CTD with blades 1 to 6 colored blue, cyan, green, yellow, orange, and red respectively. (*G*) A cartoon representation of the CTD with wrapped DNA. Residues within 4 Å of DNA are shown as van der Waals spheres. Note that GyrA box residues of each blade are interacting with the DNA minor groove.

*Escherichia coli* gyrase is a heterotetramer formed of two GyrA and two GyrB subunits (A_2_B_2_). The GyrA subunit consists of an N-terminal winged-helix domain (WHD) and Tower domain, a long coiled-coil domain, and a C-terminal β-pinwheel domain. The GyrB subunit comprises an N-terminal GHKL domain, a transducer hinge, and a topoisomerase-primase (Toprim) domain, with a species-specific insertion (Fig. 1*A*). GyrA subunits dimerize to form two interfaces called “gates”: the DNA-gate, and the C-gate. The Toprim domains of GyrB associate with GyrA to form a DNA-binding interface, while the GHKL domains are thought to be highly flexible and power DNA movements through the enzyme. While multiple crystal structures are available for isolated GHKLs and “core” (GyrB: Toprim and insertion; GyrA: WHD, Tower and coiled-coil) domains of the enzyme, there are only a handful of structural studies of full-length gyrase. Three existing cryoEM structures of *E. coli* gyrase in complex with inhibitors (7–9) display the dimerized GHKLs above the DNA-gate forming a third (ATPase) gate, whereas the crystal structure of DNA-free *Mycobacterium tuberculosis* gyrase revealed a backward-bent conformation of the GHKL domains which was proposed to be an energy-saving resting state stabilized by a species-specific (for *Corynebacteria*) insertion in the Toprim domain(10).

Gyrase is widely believed to operate by a tightly coordinated strand passage node inversion mechanism (11, 12). It is conceived that during its catalytic cycle, gyrase binds a double-stranded (ds) segment of DNA called the gate-, or G-segment across the DNA gate interface and chirally wraps ~140 bp of flanking DNA around the GyrA CTDs. Dimerization of the ATP-controlled “clamp” is proposed to capture the proximal dsDNA segment called the T- (for transported) segment in the cavity between the GHKLs. Subsequent G-segment cleavage is controlled by metal binding to the GyrB Toprim domain and involves transfer of a 5′ phosphate of each DNA chain to the corresponding catalytic tyrosine (Y122) residue within the WHD domain of GyrA. It allows the proposed opening of the enzyme, leading to the movement of the trapped T-segment through the break, inverting the node and introducing two negative supercoils. DNA can then exit the enzyme via the C-gate. The catalytic reaction of the *E. coli* enzyme was shown to require additional regulatory elements: the unstructured acidic C-terminus of GyrA (“acidic tail”) (13, 14) controlling DNA wrap, and the large insertion in the Toprim domain of the enzyme (15).

The strand passage model is supported by experiments showing that interface cross-linking prevents catalysis (16, 17); however, alternative modes of operation were proposed based on the fact that a mutant enzyme complex with only one catalytic tyrosine remains catalytically competent (18). In addition, T-segment DNA has never been visualized in any type II topoisomerase structure to date.

Here, we present high-resolution (2.3 Å) cryoEM structure of an intact, complete *E. coli* gyrase holoenzyme in the chirally wrapped state bound to a 217 bp linear DNA fragment, and the parallel structure in complex with the fluoroquinolone moxifloxacin (MFX). We describe protein–DNA interactions controlling the wrapping of DNA around the CTDs that present the T-segment DNA above and perpendicular to the G-segment. Unexpectedly, the structure shows both GHKL domains “folded down” toward the sides of the enzyme, a conformation stabilized by multiple interactions with the Toprim insert, indicating that the nucleotide binding induces a large conformational shift. GHKL dimerization, as observed in the previous AMP-PNP bound structures, is incompatible with the position of the T-segment and can only happen *after* strand passage has taken place. By examining the catalytic site in both subunits of the drug-free and MFX-bound complexes, we demonstrate that the drug-free structure is in a precleaved state distinct from those previously observed.

## In Nucleotide-Free Conditions, *E. coli* DNA Gyrase Stabilizes a Positively Supercoiled DNA Loop

To better understand the sequence of events enabling gyrase to function as a molecular motor, we conducted a systematic cryoEM investigation of the enzyme in different stages of its catalytic cycle, using a 217 bp dsDNA fragment (Mu217) from the coliphage Mu strong gyrase site (19) (SGS) that is sufficient to support effective strand passage (8, 20). The SGS is essential for phage replication by organizing phage DNA in an independent supercoiled domain within the bacterial nucleoid (21). The so-called “right arm” of the sequence is particularly important, and is hypothesized to constitute the stretch of DNA presented as a T-segment during the positively supercoiled node formation (22).

Our previous work used the gyrase-targeting toxin albicidin and nucleotide analog AMP-PNP to trap *E. coli* gyrase on Mu217 and determine the structure of the complex with cleaved DNA (8). In this structure, the DNA-binding GyrA CTD domains partially wrap DNA, and project the ends of the linear fragment at angles that are not compatible with supercoiling directionality (the projected DNA crossing occurs below the DNA-gate axis, resulting in a negatively supercoiled DNA loop). While a previous cryoEM study (7) refers to this conformation as “chirally wrapped”, we notice that in fact it is almost symmetrical and consistent with the reported Ω state that bends DNA without T-segment capture (23, 24). According to the available single-molecule and ensemble data, we anticipated that while AMP-PNP is known to release the DNA wrap, in the absence of nucleotide and low force conditions, a chirally wrapped α state predominates (23).

Following this, we collected a targeted drug and nucleotide free dataset **Gyr-Mu217**, processing of which resulted in a 2.3 Å consensus map displaying striking differences to the published gyrase structures, which are visible starting from the 2D class-averages level (Fig. 1*B*, *SI Appendix*, Table S1, Figs. S1 and S2, and Movie S1). Most notably, a linear DNA fragment was found to be fully wrapped around both CTDs forming a figure of eight-like contiguous positively supercoiled DNA loop that dominated the structure. To allow this, the CTDs of the enzyme moved upward to form a larger angle with the G-segment plane (Fig. 1 *C* and *D* and *SI Appendix*, Fig. S3). The loop was fully modeled using the available DNA sequence (*SI Appendix*, Fig. S3), and the fact that the unique Mu SGS properties position the enzyme on DNA uniformly in a defined register and orientation. This resulted in the SGS right arm chirally wrapped around one of the CTDs (CTD II) to present a T-segment for strand passage as previously established in footprinting experiments (22).The observed size of the loop is 156 bp, which is very close to the experimentally proposed values of the minimal length of DNA known to present the T-segment and thus stimulate strand passage (8, 20, 25). The wrap around the opposite CTD (CTD I) was incomplete, with the remaining nucleotides of the left arm pointing away from the enzyme. The T-segment DNA (T-DNA) is positioned ~2 nm above the G-segment DNA (G-DNA) and is almost perpendicular to it (80° angle, Fig. 1*D*).

Positively charged residues on both GyrA CTDs and Tower domains and GyrB Toprim domains delineate a conserved “guiding path” that can only accommodate one T-segment at a time (*SI Appendix*, Fig. S4 *A* and *B*). This guiding path includes a band of positive charge spiraling along the GyrA CTD that acts as a DNA-binding pulley (Fig. 1*E*).

Previous X-ray crystallography analysis established that the isolated *E. coli* gyrase CTD is an incomplete β-pinwheel domain that forms a spiral structure; this spiral was proposed to be crucial for chiral loop stabilization (26). In a previous cryoEM study with an incompletely wrapped DNA molecule (7), low resolution prevented accurate modeling of the CTD structure, as blade I was not accurately predicted by Phyre2 or Alphafold 2. In our work, we used a focused classification and refinement approach to accurately reconstruct and refine the CTD (2.9 Å) which is found to have a perfect β-pinwheel fold (27) for all 6 blades (Fig. 1*F* and *SI Appendix*, Fig. S4*C*), in full alignment with the originally published crystal structure of the *Borrelia burgdorferi* CTD. Each of the blades donates a loop that wraps around the (n-1) blade. These loops contain positively charged residues forming the so-called GyrA-box motif (28); this motif has a different degree of conservation in each blade. GyrA-box residues interact with the minor groove of DNA to stabilize five sharp bends to convey an overall ~260° bend, therefore each GyrA-box contributes ~45-60° of bend (*SI Appendix*, Fig. S4*D*). GyrA-boxes of blades 1(QRRGGKK) and 2 (TRGARGR) contain the largest number of positively charged residues concomitant with their role in interacting with the proximal (CTD II) or distal (CTD I) ends of the T-segment and maintaining it in the strand passage position (Fig. 1*G* and *SI Appendix*, Fig. S5). The GyrA-box of blade 1 is a hallmark feature of all gyrases (28–30) and is absolutely required for supercoiling and for the T-segment presentation; thus, we conclude that the observed supercoiled loop is the key precatalytic intermediate characteristic of all gyrases.

Mu phage SGS is critical for the phage DNA replication cycle and is known to bind DNA strongly and support faster supercoiling. Our model demonstrates that as was hypothesized previously (22), the right arm of Mu SGS displays AT repeats located in the minor groove facing the protein surface, while GC repeats face outward (*SI Appendix*, Figs. S3 *B* and S4 *E*). The same sequence preferences are shown by the nucleosomes (31). Strikingly, the repeated AT/GC pattern of gyrase binding can be observed on the genome level by analyzing gyrase binding site consensus sequences (32). Thus, the propensity of DNA to wrap around the CTDs controls gyrase location on DNA. The similarity with the nucleosome is further underlined by the conserved acidic tail of the CTD (not observed in our structure) which was shown to be a critical element of the *E. coli* gyrase supercoiling mechanism(13). This raises intriguing possibilities of a posttranslational modification control of gyrase activity.

We have taken advantage of the first accurate model of the DNA-bound CTD to test the function of the conserved arginines that interact with DNA and which have not been previously mutated (33). Three GyrA variants were constructed and purified: GyrA^R615Q/R617Q^, GyrA^R665Q/R667Q^, and GyrA^R712Q^. The effects of these mutations on supercoiling (which requires stabilization of a positive node), ATP-independent relaxation (which implies strand passage going in reverse, starting from an inverted node state, and expected to compete with the forward reaction) and DNA-stimulated ATPase activity of gyrase (indicative of strand passage) were measured in combination with the wild-type (WT) GyrB (*SI Appendix*, Figs. S6 *A*, S7 *A* and S8 *A*) and summarized in *SI Appendix*, Table S2. GyrA^R615Q/R617Q^ showed inhibited supercoiling and ATPase reactions but good relaxation activity. For GyrA^R665Q/R667Q^ the supercoiling inhibition was less pronounced, while GyrA^R712Q^ was not affected. However, both mutants were less active in the ATPase assay compared to the WT enzyme. Compromised writhe is also detected by the lack of the positively supercoiled DNA observed in the relaxation assays with higher concentrations of GyrA^R615Q/R617Q^ and GyrA^R665Q/R667Q^: normally, in the absence of ATP, chiral loop stabilization combined with the relaxation of the compensatory negative supercoils results in the net accumulation of positively supercoiled DNA (34). Overall, our data suggest that the mutations in the CTD where DNA enters the wrap are less important for the stability of the positive chiral loop than the ones toward the exit of the wrap and in the vicinity of the conserved GyrA-box of blade 1.

## GHKL Domains Undergo a Large Conformational Change During Catalysis

**Fig. 2.**
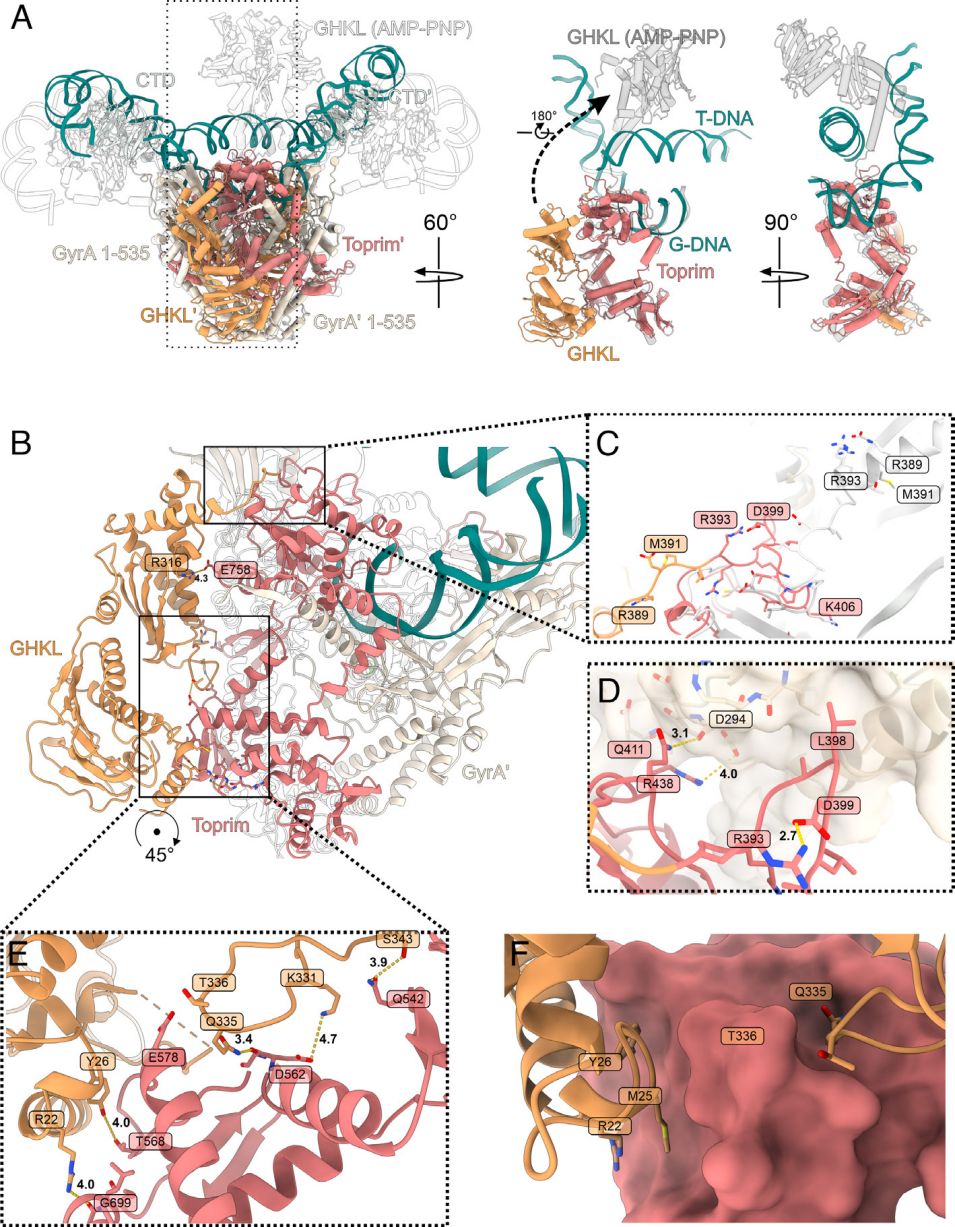
Position of the GHKL domain. (*A*) Superposition of **Gyr-Mu217** (current work, PDB: 9GBV; color scheme as before) and *E. coli* gyrase in complex with 180 bp DNA, AMP-PNP, and gepotidacin [PDB: 6RKW (7); transparent contour]. Boxed region (a single GyrB subunit) is shown in isolation on the right to illustrate the extreme motion of the GHKL (12 nm shift and 180° rotation). AMP-PNP bound GHKL is shown in gray. (*B*) An overall view of the GHKL in the downward-folded conformation. Interactions with GyrA Tower and loop conformation (*C* and *D*) and interactions with the Toprim insert (*E* and *F*) are shown as insets. AMP-PNP-bound structure [PDB: 6RKW (7)] is shown as transparent contour or white cartoon [linker comparison between **Gyr-Mu217** and PDB: 6RKW (7)].

Another central feature of the nucleotide-free structure (Fig. 2*A* and *B*) is the conformation of the GHKL domains, that are folded down such that each GHKL interacts with the Toprim insertion domain of the same GyrB subunit, reminiscent of the X-ray crystallographic structure of *M. tuberculosis* gyrase (10) or *Streptococcus pneumoniae* topoisomerase IV (35). However, both of these structures superimposed with **Gyr-Mu217** demonstrate that the T-segment sterically clashes with the conformation of the GHKLs (*SI Appendix*, Fig. S9). Therefore, it is not clear whether in those cases the “folded” conformation directly precedes supercoiling, or if it is used for the storage of an inactive enzyme as suggested (10). In contrast, it seems that the folded configuration of GHKLs in a DNA-bound state is a native feature of at least some gyrases which evolved to control coupling of ATP binding with supercoiling (23, 33, 36, 37). The docked state of the GHKLs is stabilized by a network of hydrogen bonds (Fig. 2 *B*, *E* and *F*) showing some conserved features (*SI Appendix*, Fig. S10 *A* and *B*). To test the importance of the interfaces between the GHKL and Toprim insert, we have produced variants GyrB^K331A/Q335A/T336A/S343A^ (mutations in the GHKL) and GyrB^D562A/T568A/S571A^ (mutations in the Toprim insert) and tested their activities. Variant GyrB^K331A/Q335A/T336A/S343A^ has very low supercoiling activity (*SI Appendix*, Fig. S6*B*); in line with this, both DNA-independent and DNA-stimulated ATPase activity of this mutant was virtually absent (*SI Appendix*, Fig. S8*B*) suggesting that the mutations within the critical “switch loop” of GHKL compromise the ability of this mutant to hydrolyze ATP (38–40). However, passive ATP-independent relaxation by this variant was also clearly compromised (*SI Appendix*, Fig. S7*B*). A second block of mutations (GyrB^D562A/T568A/S571A^) did not affect supercoiling (*SI Appendix*, Fig. S6*B*) but nevertheless resulted in decreased ATPase (*SI Appendix*, Fig. S8*B*) and ATP-independent relaxation activities (*SI Appendix*, Fig. S7*B*). These results show that conformational changes in the GHKL domain and interactions with the Toprim insert are necessary for the enzyme to progress between the catalytic steps even if they go downhill energetically as during the relaxation reaction.

Superposition of the chirally wrapped structure with the AMP-PNP-bound structure (PDB:6RKW (7)) demonstrates a dramatic, almost 180°, rotation and ~12 nm shift in the position of the GHKLs upon nucleotide binding (Fig. 2*A*). Importantly, the dimeric ATPase clamp which was for decades hypothesized to contain the T-segment, sterically clashes with it in the current structure (Fig. 2*A* and Movie S2). At the same time, a single GHKL subunit, if taken separately, is placed comfortably on top of the T-segment. Interestingly, this superposition places wrapped DNA in contact with the positively charged outer surface of the GHKL (*SI Appendix*, Fig. S11*A*). It also shows similarity to the observed interactions between the GHKLs and antibiotic resistance pentapeptide repeat proteins QnrB1 and MfpA (41, 42) that were shown to mimic T-segment DNA (*SI Appendix*, Fig. S11 *B* and *C*). Nevertheless, movement to this position would require each subunit to rotate and cross the path of the T-segment, and therefore would not be possible before DNA-gate opening and the T-segment moving beyond the G-segment plane.

The extreme conformational change of the GHKL is a result of the profound changes in the linker region (GyrB 386-406 in the current structure; Fig. 2 *C* and *D*). The linker residues form a loop, stabilized by a salt bridge (R393-D399) and interactions with the Tower domain of the corresponding GyrA protomer. L398 of the linker occupies a hydrophobic pocket on the GyrA surface while Q411 and R438 form hydrogen bonds to the Tower main chain (Fig. 2*D*). Contrastingly, in the AMP-PNP-bound structure the linker is 10 residues long and extends in almost the opposite direction while residues 396-386 form a part of the transducer α-helix. The linker conformation is stabilized by multiple hydrogen bonds and salt bridges as shown in Fig. 2. To test the importance of these linker residues, we have constructed a quadruple mutant GyrB^R389Q/L298A/Q441A/Q414A.^ This variant has severely decreased supercoiling, relaxation, and ATPase activities (*SI Appendix*, Table S2 and Figs. S6 *B*, S7 *B* and S8 *B*) suggesting that the mutation affects mobility of the linker and conformational movements associated with the strand passage during both supercoiling and relaxation reactions.

Residue R393 in the linker is highly conserved and forms part of the “tunnel” that directs the T-segment along the top surface of the tetramer, along with the conserved lysines of GyrA, K284, and K308, and the residues from blade 1 of the CTD (*SI Appendix*, Figs. S10 *B* and *C* and S11 *D*). Therefore, GyrB R393 could be a sensor mechanism, coupling the position of the GHKL with the position of the T-segment.

We sought to test the importance of the residues in the “T-segment tunnel” by constructing the GyrA^K284Q/R308Q/R309Q^ triple mutant, and GyrB^R393Q^ single mutant. Similarly to the CTD mutations that affected DNA wrapping, GyrA^K284Q/R308Q/R309Q^ has shown clear defects in supercoiling, and DNA-stimulated ATPase activity (*SI Appendix*, Table S2 and Figs. S6 *A* and S8 *A*), while the ATP-independent relaxation activity was not affected (*SI Appendix*, Fig. S7*B*), supporting the specific function of these residues in the T-segment presentation. Unexpectedly, GyrB^R393Q^ has higher supercoiling activity (*SI Appendix*, Fig. S6*B*), despite severely decreased ATPase activity (*SI Appendix*, Fig. S8*B*) with relaxation unaffected (*SI Appendix*, Fig. S7*B*). This result indicates that GyrB R393 may be involved in the coupling of the strand passage with ATP hydrolysis, or has another significant role in the gyrase catalytic cycle.

**Fig. 3.**
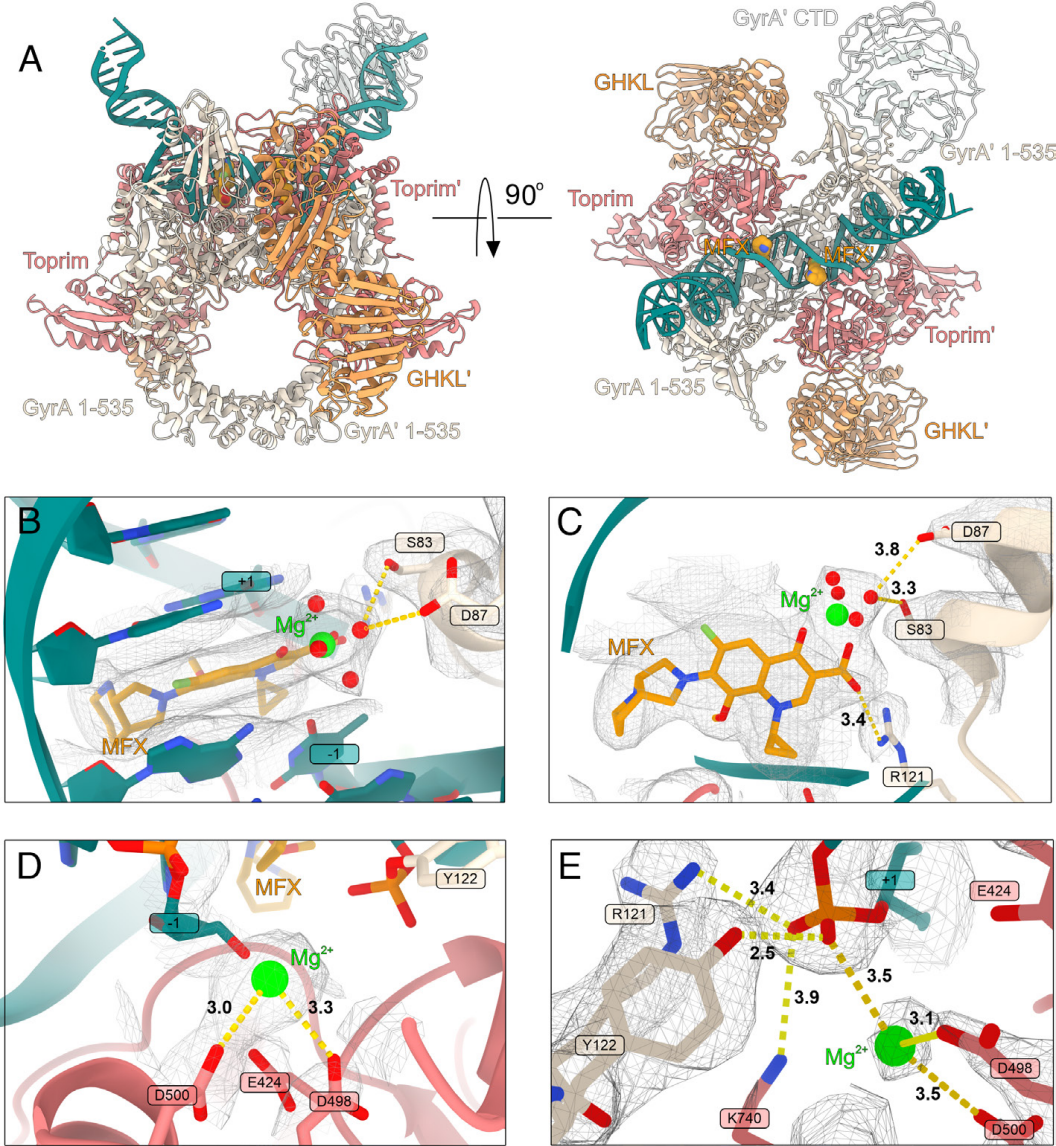
Structure of **Gyr-Mu217-MFX** and gyrase catalytic mechanism. (*A*) A cartoon representation of the **Gyr-Mu217-MFX** (current work, PDB: 9GGQ) atomic model (CTD I and chirally wrapped DNA are not modeled). MFX is shown as orange spheres. (*B*) Top view of the MFX binding pocket. MFX is shown as golden sticks and the magnesium ion as a lime sphere. Density map contoured at 15σ. GyrA residues anchoring the MFX molecule are indicated. (*C*) Side view of the MFX molecule; the water-metal ion bridge between MFX and S83/D87 of GyrA is shown. Distances in Å are indicated. Density map contoured at 9σ. (*D*) Catalytic metal ion position in the **Gyr-Mu217** structure. Distances in Å toward close residues are indicated. Density is shown contoured at 11σ. (*E*) A catalytic site in one of the GyrA protomers (chain A) in **Gyr-Mu217**. Density is shown contoured at 15σ. Catalytic residues and corresponding distances are shown. Distances in Å toward close residues are indicated. See *SI Appendix,* Fig. S14 for comparison with chain *C*.

Interestingly, a prior analysis by limited trypsin digestion suggested a special conformation stabilized by fluroquinolone (ciprofloxacin) binding that protects the GyrB 47 kDa domain (Toprim and insertion domain) from proteolysis^31^. Given that the protection has been observed only without AMP-PNP and lost upon AMP-PNP binding, we conclude that the protected conformation is likely resulting from the GHKL domains folding down to shield a large surface area of GyrB47, as observed in our structure. We hypothesized that fluroquinolones may play a role in the stabilization of the chirally wrapped state, as binding of the drug would prevent strand passage. To investigate this, we collected data on *E. coli* gyrase bound to the latest generation fluoroquinolone, MFX (**Gyr-Mu217-MFX**). This resulted in a 2.6 Å structure displaying overall the same conformation as the drug-free complex (Fig. 3*A* and *SI Appendix*, Figs. S12 and S13) with the exception of the noteworthy changes required for cleavage of DNA and intercalation of the drug. Both chains of DNA in the complex are cleaved (Fig. 3*A*) to allow intercalation of two MFX molecules per gyrase complex in symmetry-related pockets (Fig. 3*B*). A metal ion (interpreted as Mg^2+^ throughout according to the buffer composition) connects the keto acid of the fluoroquinolone with S83 and D87 of the GyrA subunit via a network of clearly visible water molecules resulting in the observed density for the Mg^2+^ ion having a characteristic octahedral shape (Fig. 3 *B* and *C*). Another contact is made by GyrA R121 from the catalytic dyad to the carboxyl of MFX (3.4 Å). The bicyclic C-7 substituent is protruding out from the DNA double helix to make contacts with E466 and K447 of GyrB: this explains previous biochemical data showing crosslinking of a chlorinated fluoroquinolone derivative to the E466C mutant (43). All three residues involved in MFX binding, S83, D87, and K447 are well described as implicated in fluoroquinolone resistance (44–46).

## Precleavage Gyrase Complex Contains a Single Metal Ion

Comparison of the catalytic centers reveals interesting differences between the MFX-bound and drug-free structures. Surprisingly, there is almost no movement of the GyrA protomers associated with DNA cleavage as for example was observed with the binding of a peptide-like drug albicidin (8). This observation helps to explain why fluoroquinolones are able to form complexes with heavily truncated enzymes (cleavage-reunion cores) and do not require DNA longer than 20 bp for stabilization (47, 48). MFX binding and associated DNA cleavage requires a shift in the position of the nucleotide, accompanied by the formation of the phosphodiester bond between Y122 and DNA from both sides of the complex. A single Mg^2+^ ion is observed next to the catalytic tyrosine coordinated by D500 and D498 of GyrB (Fig. 3*D* and *SI Appendix*, Fig. S14). In the drug-free structure, a close comparison of the Coulomb potential density between Y122 and the scissile phosphate versus between the scissile phosphate and an adjacent nucleotide allows us to discern that the two DNA chains in **Gyr-Mu217** have subtly different conformations.

Both chains were modeled as uncleaved, with the catalytic tyrosine at a 2.5 Å distance from the phosphate which is significantly closer than the equivalent 3.1 Å observed in the structure of *E. coli* gyrase with uncleaved Mu217 DNA and an allosteric cleavage inhibitor LEI-800 (9). However, while a DNA strand next to the GyrA chain A (between dA18 and dA19) could be modeled and refined well (Fig. 3*E* and *SI Appendix*, Fig. S14), the uncleaved phosphate in the antiparallel chain (between dT20 and dG21 in 5′-**T/G**ATTT-3′) fails to occupy the center of the observed density. Another difference is in the position of the metal ion coordinated by the Toprim domain acidic triad E424, D498, and D500. Two metals were previously simultaneously observed in a structure of a yeast type II topoisomerase (49), but all known structures of gyrase contain a single metal in one of the two configurations (7–9, 50). Configuration A, where the metal primarily interacts with E424, is associated with intact DNA, and was observed in complexes with the catalytic tyrosine mutated to phenylalanine, or in a complex of gyrase with the cleavage inhibitor LEI-800 (9). Configuration B, where the metal interacts with D500, was observed in complexes with cleaved DNA, including **Gyr-Mu217-MFX**. Surprisingly, while the metal in **Gyr-Mu217** chain A is bound in the B-configuration, the metal in chain C shifts away from D500 to be positioned close to D498 (2.4 Å) and to the scissile phosphate (2.7 Å), a configuration resembling a drug-free cleaved structure of *S. pneumoniae* topoisomerase IV (*SI Appendix*, Fig. S14).

Observation of a single metal bound almost exactly between the two previously observed configurations is compatible with the previously proposed mechanism where a single metal ion moves between three acidic GyrB residues^37^; however, we cannot exclude temporary recruitment of a second ion to stabilize the catalytic intermediate complex. Hence, we consider that chain C may represent an equilibrium between the precleaved DNA and the initial state of cleavage with the phosphodiester bond formed between GyrA Y122 (chain C) and dG21. The asymmetry between the chains is not surprising as it is known that DNA cleavage by type II topoisomerases is a cooperative process that happens one strand at a time (51–53) thus the **Gyr-Mu217** structure offers an insight into how this could be achieved. In the albicidin-stabilized Gyr-DNA complex (8), the **T/G** pocket is larger and is the site of the drug intercalation, hence the reason for preferential cleavage might be the preexisting stretch of DNA between these bases, as compared to the opposite strand. Given the subtle differences between the chains, and the remarkably close distance to the phosphate, we propose that the configuration we observe is very close to the actual precatalytic state. It involves stabilization of the scissile phosphate by the side chains of GyrA R121 (3.4 Å in chain A and 3.7 Å in chain C) and GyrB K740 (3.9 Å in chain A and 3.7 Å in chain C), and by the closely located single metal ion (3.5 Å in chain A and 2.7 Å in chain C). Interestingly, K740 density is less clear in the protomer where the DNA strand is fully intact. The K740A mutation was previously shown to be detrimental for enzyme activity and causes increased levels of cleavage(8). This suggests that K740 may have a particularly important role in DNA religation.

## Dimerization of ATPase Domains Controls Directionality of the Gyrase Motor

**Fig. 4.**
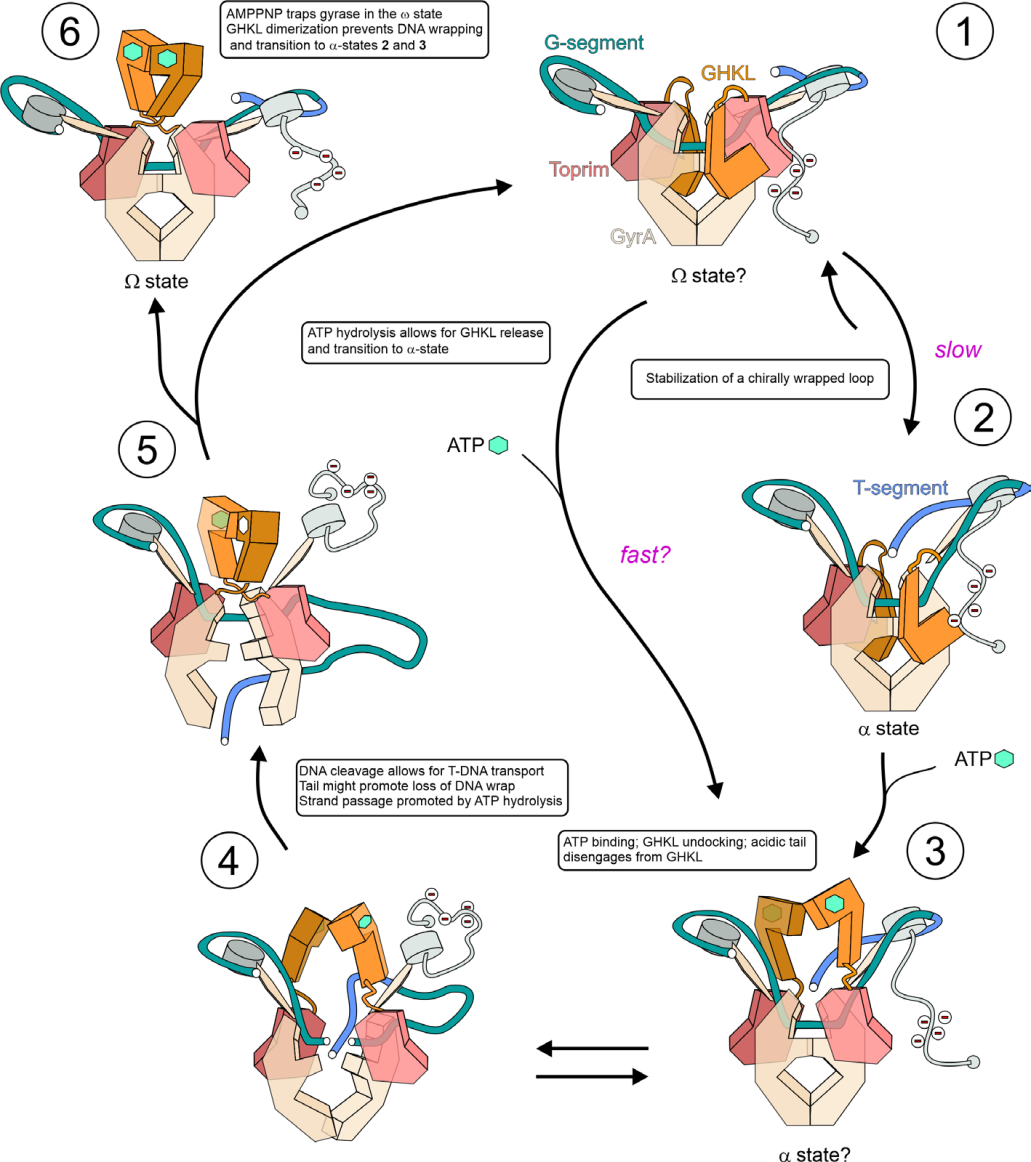
An updated model for DNA gyrase mechanism. The catalytic cycle consists of 5 stages as described in the main text. Nucleotide binding is indicated by a green hexagon (ATP) or an empty hexagon (ADP); the acidic tail of GyrA CTD is indicated by negative charges. Stage **1** – Ω-state occurring after initial DNA binding or immediately after enzyme reset. Stage **2** - α-state where DNA is constrained in a (+) supercoiled loop and the catalytic center is primed for cleavage as in **Gyr-Mu217** (PDB: 9GBV, current paper). Stage **3** – following ATP binding, the GHKL domains disengage and undergo Brownian movement toward the lowest energy conformation. They may be guided toward the T-segment by charge attraction. This hypothetical α-state may correspond to the “N-gate narrowing” observed in smFRET experiments and could be the only α-state available for some gyrases (58). Stage **4** – GHKLs follow the moving T-segment in its thermal excursion downward and prevent reversal of strand passage as it occurs. The probability of the event depends on the potential energy of DNA. GHKL movement and/or strand passage may release the acidic tail, resulting in the loss of wrap. Stepwise nucleotide hydrolysis may promote the strand passage (61). Stage **5** – after completion of strand passage, the T-segment can only escape through the bottom gate, resulting in an overall change of linking number by -2 and completion of the cycle. Hydrolysis of ATP allows for the enzyme to be reset and for the GHKLs to return to the starting position. In the case of antibiotic rescue factors QnrB1 and MfpA, this reverse motion of the GHKLs could be responsible for the release of an antibiotic from the complex. Potentially, a fast nucleotide exchange route that the enzyme can access in the presence of ATP could circumvent steps 1 to 2 to yield the α-state sooner. Stage **6** – a trapped poststrand passage Ω-state with the dimerized GHKL module as seen in the AMP-PNP bound cryoEM structures occurs when the enzyme reset cannot happen. DNA may or may not be cleaved depending on the stabilizing agent present.

According to the node inversion mechanism proposed more than 40 y ago (11, 12), the directionality of gyrase results from the chiral selection, and the input of energy from ATP that is used to drive unidirectional strand passage. In this work, we have determined the molecular mechanism responsible for this chiral selection and showed that without ATP, gyrase indeed stabilizes a positively supercoiled DNA loop. We propose the updated model for the gyrase molecular motor illustrated in Fig. 4.

Initial binding of gyrase to DNA may result in one of the Ω states, which are not chirally wrapped (stage **1**) and were visualized previously (7–9). The position of the GHKL domains in this state is unknown, but based on our structure and the mutant data it is clear that, at least in the case of *E. coli* gyrase and in the absence of the nucleotide, they interact with the Toprim insert subdomains to allow for chiral wrap stabilization and T-segment trapping to yield the initial α state (**Gyr-Mu217**; stage **2**). This GHKL “folding” mechanism might have evolved to increase coupling in gyrase by preventing dimerization and ATP hydrolysis in the absence of the T-segment (24). The chiral wrap occurs with high efficiency on all substrates (linear, negatively, and positively supercoiled DNA)(54) and is enabled by tight binding of DNA to the β-pinwheels; the wrapped state has been shown to be well-populated in rotor bead tracking experiments in the absence of nucleotide (23). A Mu217 sequence might be particularly conducive for wrapping, facilitating α-state formation in the absence of the nucleotide. To enable progressive negative supercoiling on a substrate of increasing superhelicity, DNA binding to CTDs is necessarily tight. It has been shown that an artificial increase in CTD positive charge stabilizes the wrap, allowing for a small increase in maximal superhelicity, but at the expense of a large decrease in supercoiling speed (33). Thus it is the transition to the next stage that ultimately requires energy input in the form of ATP binding (55). After binding of ATP, conformational changes around the active site (previously described in the literature as rearrangement of the switch loop, *SI Appendix*, Fig. S15) result in an 11° rotation of the transducer domain(38–40, 56, 57) that may be sufficient for disengagement and undocking of the GHKLs leading to stage **3**. It was shown in single-molecule experiments that in the presence of ATP, gyrase can access a “fast” pathway to the ATP-bound α-state (3) which may or may not skip step **2**. We imagine the ATP-bound state **3** as having the GHKLs not yet dimerized/rotated but already interacting with the trapped T-segment. smFRET experiments on *Bacillus subtilis* gyrase reported N-gate “narrowing” distinct from dimerization and was observed only in conjunction with T-segment presentation (58).

It may be that in Gram-positive enzymes that do not have the Toprim insert, the nucleotide-free α-state is also similar to **3**. The energy of ATP binding ultimately enables a conformational transition to the next lower potential well where the GHKLs are dimerized and rotated 180° as observed in the AMP-PNP-bound structure (stage **4 to 5**). This relaxation requires the T-segment to move beyond the plane of the G-segment, which in turn requires DNA cleavage. It has been shown that gyrase naturally maintains an equilibrium between cleaved and intact DNA which is normally shifted toward religation; however, nucleotide binding stimulates DNA cleavage presumably by promoting strand passage (34, 53, 54, 59). The T-segment passage and conversion to the product state necessitates disruption of the original wrapped complex (33) that is observed upon nucleotide binding (60). The mechanism for this loss of wrap might include a conserved acidic tail in GyrA which was shown to be essential for *E. coli* gyrase supercoiling (13). While the tail is unstructured, and not observed in our maps, we propose that its initial position (which may involve interaction with docked GHKLs) allows the CTDs to move upward and fully engage DNA, stabilizing a positive supercoil. The events associated with the nucleotide binding could simultaneously disengage the acidic tail, which in turn facilitates the removal of wrapped DNA from the CTD. Thus, effective supercoiling would require tight coordination of nucleotide binding with both the loss of wrap and DNA cleavage to create a brief window of opportunity during which strand passage can happen. The molecular basis of this coordination is not determined and is of considerable interest.

As the probability of strand passage per round of nucleotide binding depends on the nature of the substrate (e.g. almost 1 for positively supercoiled DNA and almost 0 for highly negatively supercoiled DNA following incubation with AMP-PNP) (37), in our preferred model it is a random event, the likelihood of which depends on temperature and the DNA torsional energy. We do not favor but acknowledge the possibility of other models with an active role for the GHKLs in moving the T-segment downward. After a successful strand passage attempt resulting in the T-segment hovering below the DNA-gate plane, the GHKLs can fully rotate (stage **5**) and dimerize, leading to the Ω conformation (stage **1**). Rotation and dimerization prevent the reversal of the strand passage process (upward escape of the T-segment) ensuring reaction directionality. With AMP-PNP, the dimer remains irreversibly locked and is observed as such by cryoEM (stage **6**), while in the course of a normal reaction, dimerization promotes ATP hydrolysis that could be important for fast strand passage completion (61). Hydrolysis of ATP by a monomeric GHKL is slow and ensures that the enzyme has multiple attempts at strand passage even on negatively supercoiled substrates. It has been shown that an enzyme that is incapable of cleaving DNA does not demonstrate a DNA-stimulated increase in the rate of ATPase activity, supporting the proposed sequence of events (54). It is also noteworthy that binding of the peptide inhibitors albicidin or microcin B17 requires a strand passage attempt and the binding (but not hydrolysis) of the nucleotide; at the same time, hydrolysis of the nucleotide was shown to be important for the activity of antibiotic resistance factors QnrB1 and MfpA (41, 42). While the peptide toxins could occupy the larger space between dissociated GyrA protomers occurring after strand passage, the resistance factors perhaps use the motion of relaxing GHKLs to remove bound drugs (*SI Appendix*, Fig. S11). Following ATP hydrolysis the GHKLs return to their initial folded conformation.

A crucial difference between our model and previously proposed schemes (e.g. (62–65)) is the observation that the GHKL dimerization and rotation captured by AMP-PNP-containing structures can only happen after the strand passage has taken place: the observed position of the T-segment in our structure is sterically incompatible with the required rotation of the GHKLs (Movie S2). For example, a report on a crystal structure of the dimerized GHKL clamp in *S. pneumoniae* topoisomerase IV (PDB:5J5Q) suggests that the 14 bp DNA found to interact with the inside of the clamp is representative of a bona fide T-segment capture (65). However, as dimerization and rotation of the clamp is a conserved feature for type II topoisomerases (66, 67) it is unlikely that this structure directly represents one of the T-segment bound states on the catalytic pathway. Nevertheless, the GHKLs can still clamp the T-segment without rotation as depicted in step **3** (N-gate narrowing stage). Thus, the residues and DNA-binding interfaces interrogated in the abovementioned report (65) are likely still important in progression from stage **3** to stage **5** (the ATP-bound α-state) but it is the GHKL rotation and dimerization that acts as an irreversible conformation change (a “pawl”), ensuring the overall directionality.

Our model allows us to make important predictions regarding the sequence of events and role of individual gyrase subunits and interfaces. Verifying these would require further data on the structures of different α-states for different type II topoisomerases and the application of noninterfering, in solution techniques such as smFRET (68) or EPR to directly observe predicted conformational changes. These experiments could also investigate the proposed key role of the acidic C-tail in movement between different conformational stages, and its suggested role in setting supercoiling set point (69). We believe that the structural and theoretical framework proposed in this manuscript will promote fruitful discussions toward the fundamental understanding and practical use of gyrase and other molecular motors.

## Methods

### Protein Purification and CryoEM Sample Preparation.

*E. coli* GyrA and GyrB proteins were purified as previously described (8) using metal affinity, Strep-tag, and ion-exchange chromatography. To produce the described GyrA and GyrB variants, mutations were introduced into pET28-GyrATS and pET28-GyrBTS by the two-fragment assembly protocol (70) using NEBuilder HiFi assembly kit (8). To prepare cryoEM samples, proteins were concentrated to 12 mg/mL prior to complex formation and dialyzed overnight at 4 °C into cryo-EM buffer (25 mM Na-HEPES pH 8, 30 mM potassium acetate, 2.5 mM magnesium acetate, 0.5 mM Tris [2-carboxyethyl) phosphine (TCEP)] in the presence of equimolar amount of Mu217 DNA in a Pur-A-Lyzer Mini (Merck). Mu217 DNA was purified as previously described (8). For the MFX complex, drug was added to the dialysis buffer at 50 µM. Dialyzed sample was concentrated to 15 µM, additionally supplemented with 100 µM MFX and incubated at 37 °C for 15 min. Before grid freezing, CHAPSO (8 mM) was added and the samples were centrifuged at 21,000×*g* at 4 °C for 60 min. 4 µL of sample was applied to Quantifoil (R2/1, 300 copper mesh) glow-discharged grids. Grids were blotted for 6 s and plunge-frozen in liquid ethane using a Vitrobot Mark IV (Thermo; at 95% humidity, 10 °C).

### CryoEM Data Collection and Analysis.

CryoEM data were collected on a Krios G3i microscope at the Polish National cryoEM Facility, SOLARIS, using a Gatan K3 camera with a Gatan BioQuantum energy filter operated with a slit width of 20 eV. Movies were collected at a 10,5000× nominal magnification, resulting in a calibrated physical pixel size of 0.86 Å using EPU v2.10.0.1941REL. Movies were saved at physical pixel size as gain-corrected TIFF files. For **Gyr-Mu217**, 8,508 movies were collected with the range of defoci set as −2.1, −1.8, −1.5, −1.2, −0.9 μm and a total dose of 41.84 e/Å^2^ over 40 frames. 8,405 movies were kept for further processing in CryoSPARC v. 4.2.1 (71). Movies were motion and CTF corrected in patch mode. 65,8859 particles were picked using cryoSPARC template picker and extracted with a pixel size of 1.72 Å/px. Binned particles underwent two rounds of 2D classification to yield a cleaned stack of 230,444 particles. The ab initio job was used to classify in 3D, followed by a nonuniform refinement (72). Particles were re-extracted at physical pixel size and refined correcting for local defocus yielding a 2.3 Å consensus map. Particles underwent a round of reference-based motion correction (73) as implemented in cryoSPARC, followed by heterogenous refinement with two classes (a map and a low-passed filtered map) to remove particles that did not contribute to high resolution structure. After a second round of polishing, 3^rd^ and 4^th^ order CTF aberrations correction (74) and Ewald sphere correction (75), the final resolution was 2.32 Å after nonuniform refinement in cryoSPARC. To further improve density for CTD II in the map, 3D classification without alignment was carried out with 10 classes, using a mask around the CTD. Local refinement of particles from the three best classes yielded a 2.94 Å map which was combined with the consensus map using ChimeraX *fit in map* tool and the *vop maximum* command to obtain a composite map used for refinement. The same approach was used to obtain local maps for CTD I, CTD II+T-DNA, and both GHKL domains as depicted in *SI Appendix*, Fig. S1. For **Gyr-Mu217-MFX**, 4 500 movies were collected using the range of defoci set as −2.1, −1.8, −1.5, −1.2, −0.9 μm, and a total dose of 40.68 e/Å^2^ over 40 frames. 4,246 were kept for further processing. 19,0069 particles were picked using Topaz (76) and extracted with a pixel size of 1.72 Å/px. Binned particles underwent a round of 2D classification yielding 15,2001 particles, and a round of 3D classification (Ab initio job) yielding 13,3625 particles. After reextraction, refinement, and a reference-based local motion correction as implemented in cryoSPARC 4.4, followed by a nonuniform refinement with correcting for local defocus, 3rd and 4th order CTF aberrations and Ewald sphere correction, a map was obtained with a resolution of 2.46 Å used for the initial refinement and building of the cleavage-reunion core region (GyrA 7 to 524; GyrB 405 to 804). This map displayed heterogeneity in the position of GyrA CTDs and GyrB GHKL domains; to address this, a mask was applied around the GHKL domains (*SI Appendix*, Fig. S12) followed by classification without alignment in cryoSPARC (5 classes). Classes with GHKL density predominantly from one side or the other of the core complex were obtained; symmetrical classes were combined and refined to yield a 2.61 Å consensus map used for building of the model that incorporated the GyrB GHKLs and the better resolved GyrA CTD domain (CTD II). Because of the lack of observable differences between the positions of wrapped DNA and the CTDs between the **Gyr-Mu217** and **Gyr-Mu217-MFX** no local refinements were carried out in the latter case.

### Model Building and Refinement.

The model for the cleavage-reunion core was manually built in Coot(77) based on the previously available high-resolution structure [PDB: 7Z9C (8)]. The GHKL domain was manually built based on the available crystal structure [PDB: 1EI1 (38)]. The C-terminal domain was built using the crystal structure PDB: 1ZI0 (26) and ModelAngelo(78) followed by manual geometry optimization in Coot. Poorly resolved regions were refined using ISOLDE (79). To build DNA, bases around the cleavage site were manually assigned and the rest of the wrapped DNA was constructed using ideal B-form DNA blocks in Coot. A cryoREAD-generated model (80) was used for guidance and to verify DNA positioning. The complete model was refined in real space using Phenix (81) against an unsharpened map using Ramachandran restraints and secondary structure restraints for protein and DNA bases. NCS restraints were used during the first few rounds of refinement for stabilization and subsequently switched off. All visualization, superpositions, and surface calculations were done in ChimeraX (82). To build the **Gyr-MFX-Mu217** model, GHKL domains and CTD II were copied from **Gyr-Mu217**, rigid-body fitted, and refined in real space against the consensus map. As the **Gyr-Mu217-MFX** map displayed no differences in the positions of the wrapped DNA and CTDs compared to **Gyr-Mu217**, only one CTD and the better-resolved 42-bp region around the active site were built.

### ATPase Activity Assays.

ATPase assays were carried out using a PK/LDH linked assay (83). DNA gyrase complex (50 nM) or GyrB alone (50 nM) were mixed in assay buffer (50 mM Tris–Cl pH 7.5, 1 mM EDTA, 5 mM MgCl_2_, 5 mM DTT, 10% (w/v) glycerol, 0.8 mM PEP, 0.4 mM NADH, and 1 U of PK/LDH mix (Sigma) with 10.5 nM linear pBR322 DNA (if present) in a 100 µL final reaction volume. Reactions were started by addition of 2 mM ATP. Absorbance at 340 nm was measured for 60 min in 1-min increments at 25 °C using a plate reader (Tecan). One NADH is oxidized for each ATP hydrolyzed, allowing for simple calculation of ATP hydrolysis rates (extinction coefficient 6.22 mM^−1^cm^−1^). Novobiocin (5 µM) was used as a control for inhibition of gyrase-specific ATPase activity.

### DNA Supercoiling and Relaxation Activity Assays.

Purified gyrase subunits were mixed in an equimolar ratio and incubated on ice. The complex was diluted to 1 µM using HGED buffer (50 mM Na-HEPES pH 8, 10 % glycerol, 1 mM EDTA, 2 mM DTT). Supercoiling reactions were assembled in 30 µL where DNA gyrase was mixed in buffer containing 35 mM Tris-HCl pH 7.5, 24 mM KCl, 4 mM MgCl_2_, 2 mM DTT, 1.8 mM spermidine, 1 mM ATP, 6.0% (w/v) glycerol, and 0.1 mg/mL BSA with 500 µg of relaxed pBR322 DNA (Inspiralis). Reactions were incubated for 30 min at 37 °C and then stopped using 30 µL chloroform:isoamyl alcohol (24:1 v/v) and 30 µL of STEB (40% (w/v) sucrose, 100 mM Tris-HCl pH 8, 10 mM EDTA, 0.5 mg/mL bromophenol blue).

For relaxation assays, the gyrase complex was prepared in the same way and incubated with negatively supercoiled pBR322 DNA (Inspiralis) in reaction buffer (35 mM Tris-HCl pH 7.5, 24 mM KCl, 4 mM MgCl_2,_ 2 mM DTT, 6% glycerol, and 0.1 mg/mL BSA) for 60 min at 37 °C and stopped in the same way as for supercoiling assay.

All stopped reaction samples were centrifuged at 14,000×*g* for 1 min and the aqueous phase was loaded onto a 1% TAE (40 mM Tris base, 20 mM acetic acid and 1 mM EDTA) or 1% TBE (89 mM Tris base, 89 mM boric acid, 2 mM EDTA) agarose gel and run at 80 V for 2 h in TAE or TBE buffer. Once complete, gels were stained with ethidium bromide (10 µg mL^−1^) for 15 min, destained with water, and visualized using a ChemiDoc imaging system (Bio-Rad). Concentrations of enzyme used in the assay are stated in the figures (supercoiling and relaxation) or figure legends (ATPase).

## Supplementary Material

Appendix 01 (PDF)

Movie S1. **Overall structure of Gyr-Mu217**. The movie starts by showing the composite cryoEM map for **Gyr-Mu217** (EMD-51222) colored according to the protein domains as in **Figure 1**. The map transitions into a cartoon atomic model (PDB: 9GBV). The protein part of the model fades to show the chiral DNA loop in isolation with T-DNA colored in blue and G-DNA in green.

Movie S2. **Comparison of PDB: 6RKW and Gyr-Mu217**. The movie starts by showing a chirally wrapped DNA loop from PDB: 9GBV (α-state) with G-DNA colored green and T-DNA blue. The DNA fragment from PDB: 6RKW (Ω state) is superimposed in light gray. The dimerised AMP-PNP bound GyrB subunits from the same model (colored pink and gray) are shown to illustrate the clash with the position of T-DNA. Finally, positions of the single GyrB subunit from the two models are compared, PDB: 6RKW colored gray and **Gyr-Mu217** colored pink and orange according to the color scheme in **Figure 1** and throughout the manuscript. The large shift and rotation of the GHKL domain is visible, compared to the almost identical position of the Toprim insertion domain.

## Data Availability

All data needed to evaluate the conclusions in the paper are present in the paper or available from the following public repositories. The **Gyr-Mu217** and **Gyr-Mu217-MFX** coordinates have been submitted to the Protein Data Bank (https://www.rcsb.org/) with PDB IDs 9GBV and 9GGQ, respectively. Corresponding EM maps have been submitted to the Electron Microscopy Data Bank (https://www.ebi.ac.uk/pdbe/emdb/) with IDs EMD-51222 (composite map) and EMD-51339, respectively. The consensus EM map for **Gyr-Mu217** has been submitted to the EMDB with the ID EMD-51218; focused EM maps have been submitted to EMDB with the IDs EMD-51211, EMD-51212, EMD-51213, EMD-51215, EMD-51216 (details available in *SI Appendix*, Fig. S1). Raw EM data are uploaded to the EMPIAR repository (84) (https://www.ebi.ac.uk/empiar) under the IDs EMPIAR-12230 (**Gyr-Mu217** dataset) and EMPIAR-12255 (**Gyr-Mu217-MFX** dataset).

## References

[1] W. Hwang, M. Karplus, Structural basis for power stroke vs. Brownian ratchet mechanisms of motor proteins. Proc. Natl. Acad. Sci. U.S.A. **116**, 19777–19785 (2019). 31506355 10.1073/pnas.1818589116 PMC6778177

[2] R. Dutta, M. Inouye, GHKL, an emergent ATPase/kinase superfamily. Trends Biochem. Sci. **25**, 24–28 (2000). 10637609 10.1016/s0968-0004(99)01503-0

[3] A. Koch , MORC proteins: Novel players in plant and animal health. Front. Plant Sci. **8**, 1720 (2017). 29093720 10.3389/fpls.2017.01720 PMC5651269

[4] Y. Gu Bacterial Shedu immune nucleases share a common enzymatic core regulated by diverse sensor domains. bioRxiv[Preprint] (2023). 10.1101/2023.08.10.552793 (Accessed 10 August 2023). PMC11805627 39742666

[5] S. M. Vos, E. M. Tretter, B. H. Schmidt, J. M. Berger, All tangled up: How cells direct, manage and exploit topoisomerase function. Nat. Rev. Mol. Cell Bio. **12**, 827–841 (2011). 22108601 10.1038/nrm3228 PMC4351964

[6] N. G. Bush, I. Diez-Santos, L. R. Abbott, A. Maxwell, Quinolones: Mechanism, lethality and their contributions to antibiotic resistance. Molecules **25**, 5662 (2020). 33271787 10.3390/molecules25235662 PMC7730664

[7] A. Vanden Broeck, C. Lotz, J. Ortiz, V. Lamour, Cryo-EM structure of the complete DNA gyrase nucleoprotein complex. Nat. Commun. **10**, 4935 (2019). 31666516 10.1038/s41467-019-12914-y PMC6821735

[8] E. Michalczyk , Molecular mechanism of topoisomerase poisoning by the peptide antibiotic albicidin. Nat. Catalysis **6**, 52-67 (2023), 10.1038/s41929-022-00904-1. PMC9886550 36741192

[9] A. T. Bakker , Discovery of isoquinoline sulfonamides as allosteric gyrase inhibitors with activity against fluoroquinolone-resistant bacteria. Nat. Chem. **16**, 1462-1472 (2024), 10.1038/s41557-024-01516-x. 38898213 PMC11374673

[10] S. Petrella , Overall structures of DNA gyrase reveal the role of a GyrB-specific insert in ATPase activity. Structure **27**, 579 (2019). 30744994 10.1016/j.str.2019.01.004

[11] K. Mizuuchi, L. M. Fisher, M. H. Odea, M. Gellert, DNA gyrase action involves the introduction of transient double-strand breaks into DNA. Proc. Natl. Acad. Sci.-Biol. **77**, 1847–1851 (1980). 10.1073/pnas.77.4.1847 PMC348605 6246508

[12] P. O. Brown, N. R. Cozzarelli, A sign inversion mechanism for enzymatic supercoiling of DNA. Science **206**, 1081–1083 (1979). 227059 10.1126/science.227059

[13] E. M. Tretter, J. M. Berger, Mechanisms for defining supercoiling set point of DNA gyrase orthologs: I. A nonconserved acidic C-terminal tail modulates Escherichia coli gyrase activity. J. Biol. Chem. **287**, 18636–18644 (2012). 22457353 10.1074/jbc.M112.345678 PMC3365713

[14] M. A. Lanz, M. Farhat, D. Klostermeier, The acidic C-terminal tail of the GyrA subunit moderates the DNA supercoiling activity of Bacillus subtilis gyrase. J. Biol. Chem. **289**, 12275–12285 (2014). 24563461 10.1074/jbc.M114.547745 PMC4007426

[15] A. J. Schoeffler, A. P. May, J. M. Berger, A domain insertion in GyrB adopts a novel fold that plays a critical role in gyrase function. Nucleic Acids Res. **38**, 7830–7844 (2010). 20675723 10.1093/nar/gkq665 PMC2995079

[16] N. L. Williams, A. Maxwell, Probing the two-gate mechanism of DNA gyrase using cysteine cross-linking. Biochemistry **38**, 13502–13511 (1999). 10521257 10.1021/bi9912488

[17] N. L. Williams, A. J. Howells, A. Maxwell, Locking the ATP-operated clamp of DNA gyrase: Probing the mechanism of strand passage. J. Mol. Biol. **306**, 969–984 (2001). 11237612 10.1006/jmbi.2001.4468

[18] A. Gubaev, D. Weidlich, D. Klostermeier, DNA gyrase with a single catalytic tyrosine can catalyze DNA supercoiling by a nicking-closing mechanism. Nucleic Acids Res. **44**, 10354–10366 (2016). 27557712 10.1093/nar/gkw740 PMC5137430

[19] M. L. Pato, M. M. Howe, N. P. Higgins, A DNA gyrase-binding site at the center of the bacteriophage Mu genome is required for efficient replicative transposition. Proc. Natl. Acad. Sci. U.S.A. **87**, 8716–8720 (1990). 2174162 10.1073/pnas.87.22.8716 PMC55030

[20] O. A. Pierrat, A. Maxwell, Evidence for the role of DNA strand passage in the mechanism of action of microcin B17 on DNA gyrase. Biochemistry **44**, 4204–4215 (2005). 15766248 10.1021/bi0478751

[21] M. L. Pato, M. Banerjee, The Mu strong gyrase-binding site promotes efficient synapsis of the prophage termini. Mol. Microbiol. **22**, 283–292 (1996). 8930913 10.1046/j.1365-2958.1996.00115.x

[22] M. Oram, A. A. Travers, A. J. Howells, A. Maxwell, M. L. Pato, Dissection of the bacteriophage Mu strong gyrase site (SGS): Significance of the SGS right arm in Mu biology and DNA gyrase mechanism. J. Bacteriol. **188**, 619–632 (2006). 16385052 10.1128/JB.188.2.619-632.2006 PMC1347280

[23] A. Basu , Dynamic coupling between conformations and nucleotide states in DNA gyrase. Nat. Chem. Biol. **14**, 565 (2018). 29662209 10.1038/s41589-018-0037-0 PMC10121156

[24] A. Basu, A. J. Schoeffler, J. M. Berger, Z. Bryant, ATP binding controls distinct structural transitions of DNA gyrase in complex with DNA. Nat. Struct. Mol. Biol. **19**, 538–U105 (2012). 22484318 10.1038/nsmb.2278 PMC5660678

[25] S. E. Critchlow , The interaction of the F plasmid killer protein, CcdB, with DNA gyrase: Induction of DNA cleavage and blocking of transcription. J. Mol. Biol. **273**, 826–839 (1997). 9367775 10.1006/jmbi.1997.1357

[26] A. J. Ruthenburg, D. M. Graybosch, J. C. Huetsch, G. L. Verdine, A superhelical spiral in the DNA gyrase A C-terminal domain imparts unidirectional supercoiling bias. J. Biol. Chem. **280**, 26177–26184 (2005). 15897198 10.1074/jbc.M502838200

[27] K. D. Corbett, R. K. Shultzaberger, J. M. Berger, The C-terminal domain of DNA gyrase A adopts a DNA-bending β-pinwheel fold. Proc. Natl. Acad. Sci. U.S.A. **101**, 7293–7298 (2004). 15123801 10.1073/pnas.0401595101 PMC409912

[28] V. M. Kramlinger, H. Hiasa, The “GyrA-box” is required for the ability of DNA gyrase to wrap DNA and catalyze the supercoiling reaction. J. Biol. Chem. **281**, 3738–3742 (2006). 16332690 10.1074/jbc.M511160200

[29] M. A. Lanz, D. Klostermeier, The GyrA-box determines the geometry of DNA bound to gyrase and couples DNA binding to the nucleotide cycle. Nucleic Acids Res. **40**, 10893–10903 (2012). 22977179 10.1093/nar/gks852 PMC3510516

[30] E. M. Tretter, J. C. Lerman, J. M. Berger, A naturally chimeric type IIA topoisomerase in *Aquifex aeolicus* highlights an evolutionary path for the emergence of functional paralogs. Proc. Natl. Acad. Sci. U.S.A. **107**, 22055–22059 (2010). 21076033 10.1073/pnas.1012938107 PMC3009783

[31] K. Luger, A. W. Mader, R. K. Richmond, D. F. Sargent, T. J. Richmond, Crystal structure of the nucleosome core particle at 2.8 angstrom resolution. Nature **389**, 251–260 (1997). 9305837 10.1038/38444

[32] D. Sutormin, N. Rubanova, M. Logacheva, D. Ghilarov, K. Severinov, Single-nucleotide-resolution mapping of DNA gyrase cleavage sites across the *Escherichia coli* genome. Nucleic Acids Res. **47**, 1373–1388 (2019). 30517674 10.1093/nar/gky1222 PMC6379681

[33] M. J. Hobson, Z. Bryant, J. M. Berger, Modulated control of DNA supercoiling balance by the DNA-wrapping domain of bacterial gyrase. Nucleic Acids Res. **48**, 2035–2049 (2020). 31950157 10.1093/nar/gkz1230 PMC7038939

[34] P. F. Chan , Thiophene antibacterials that allosterically stabilize DNA-cleavage complexes with DNA gyrase. Proc. Natl. Acad. Sci. U.S.A. **114**, E4492–E4500 (2017). 28507124 10.1073/pnas.1700721114 PMC5465892

[35] I. Laponogov , Structure of an “open” clamp type II topoisomerase-DNA complex provides a mechanism for DNA capture and transport. Nucleic Acids Res. **41**, 9911–9923 (2013). 23965305 10.1093/nar/gkt749 PMC3834822

[36] A. Sugino, N. R. Cozzarelli, The Intrinsic Atpase of DNA Gyrase. J. Biol. Chem. **255**, 6299–6306 (1980). 6248518

[37] A. D. Bates, M. H. O’Dea, M. Gellert, Energy coupling in *Escherichia coli* DNA gyrase: The relationship between nucleotide binding, strand passage, and DNA supercoiling. Biochemistry **35**, 1408–1416 (1996). 8634270 10.1021/bi952433y

[38] D. B. Wigley, G. J. Davies, E. J. Dodson, A. Maxwell, G. Dodson, Crystal-structure of an N-terminal fragment of the DNA gyrase B-protein. Nature **351**, 624–629 (1991). 1646964 10.1038/351624a0

[39] C. Ban, M. Junop, W. Yang, Transformation of MutL by ATP binding and hydrolysis: A switch in DNA mismatch repair. Cell **97**, 85–97 (1999). 10199405 10.1016/s0092-8674(00)80717-5

[40] K. D. Corbett, J. M. Berger, Structural dissection of ATP turnover in the prototypical GHL ATPase topoVI. Structure **13**, 873–882 (2005). 15939019 10.1016/j.str.2005.03.013

[41] L. Mazurek , Pentapeptide repeat protein QnrB1 requires ATP hydrolysis to rejuvenate poisoned gyrase complexes. Nucleic Acids Res. **49**, 1581–1596 (2021). 33434265 10.1093/nar/gkaa1266 PMC7897471

[42] L. P. Feng , The pentapeptide-repeat protein, MfpA, interacts with mycobacterial DNA gyrase as a DNA T-segment mimic. Proc. Natl. Acad. Sci. U.S.A. **118**, e2016705118 (2021). 33836580 10.1073/pnas.2016705118 PMC7980463

[43] A. Mustaev , Fluoroquinolone-gyrase-DNA complexes TWO modes of drug binding. J. Biol. Chem. **289**, 12300–12312 (2014). 24497635 10.1074/jbc.M113.529164 PMC4007428

[44] H. Yoshida, M. Bogaki, M. Nakamura, S. Nakamura, Quinolone resistance-determining region in the DNA gyrase gyrA gene of Escherichia coli. Antimicrob. Agents Chemother. **34**, 1271–1272 (1990). 2168148 10.1128/aac.34.6.1271 PMC171799

[45] H. Yoshida, M. Bogaki, M. Nakamura, L. M. Yamanaka, S. Nakamura, Quinolone resistance-determining region in the DNA gyrase gyrB gene of *Escherichia coli*. Antimicrob. Agents Chemother. **35**, 1647–1650 (1991). 1656869 10.1128/aac.35.8.1647 PMC245234

[46] J. Heddle, A. Maxwell, Quinolone-binding pocket of DNA gyrase: Role of GyrB. Antimicrob. Agents Chemother. **46**, 1805–1815 (2002). 12019094 10.1128/AAC.46.6.1805-1815.2002 PMC127264

[47] M. E. Cove, A. P. Tingey, A. Maxwell, DNA gyrase can cleave short DNA fragments in the presence of quinolone drugs. Nucleic Acids Res. **25**, 2716–2722 (1997). 9207016 10.1093/nar/25.14.2716 PMC146802

[48] H. Gmunder, K. Kuratli, W. Keck, In the presence of subunit A inhibitors DNA gyrase cleaves DNA fragments as short as 20 bp at specific sites. Nucleic Acids Res. **25**, 604–610 (1997). 9016602 10.1093/nar/25.3.604 PMC146451

[49] B. H. Schmidt, A. B. Burgin, J. E. Deweese, N. Osheroff, J. M. Berger, A novel and unified two-metal mechanism for DNA cleavage by type II and IA topoisomerases. Nature **465**, 641–U139 (2010). 20485342 10.1038/nature08974 PMC2882514

[50] B. D. Bax, G. Murshudov, A. Maxwell, T. Germe, DNA topoisomerase inhibitors: Trapping a DNA-cleaving machine in motion. J. Mol. Biol. **431**, 3427–3449 (2019). 31301408 10.1016/j.jmb.2019.07.008 PMC6723622

[51] S. C. Kampranis, A. Maxwell, The DNA gyrase-quinolone complex - ATP hydrolysis and the mechanism of DNA cleavage. J. Biol. Chem. **273**, 22615–22626 (1998). 9712890 10.1074/jbc.273.35.22615

[52] E. L. Zechiedrich, K. Christiansen, A. H. Andersen, O. Westergaard, N. Osheroff, Double-stranded DNA cleavage religation reaction of eukaryotic topoisomerase-Ii - evidence for a nicked DNA intermediate. Biochemistry **28**, 6229–6236 (1989). 2551367 10.1021/bi00441a014

[53] F. Mueller-Planitz, D. Herschlag, Coupling between ATP binding and DNA cleavage by DNA topoisomerase II - A unifying kinetic and structural mechanism. J. Biol. Chem. **283**, 17463–17476 (2008). 18403371 10.1074/jbc.M710014200 PMC2427340

[54] S. C. Kampranis, A. D. Bates, A. Maxwell, A model for the mechanism of strand passage by DNA gyrase. Proc. Natl. Acad. Sci. U.S.A. **96**, 8414–8419 (1999). 10411889 10.1073/pnas.96.15.8414 PMC17530

[55] A. Sugino, N. P. Higgins, P. O. Brown, C. L. Peebles, N. R. Cozzarelli, Energy coupling in DNA gyrase and mechanism of action of novobiocin. Proc. Natl. Acad. Sci. U.S.A. **75**, 4838–4842 (1978). 368801 10.1073/pnas.75.10.4838 PMC336216

[56] V. Lamour, L. Hoermann, J. M. Jeltsch, P. Oudet, D. Moras, An open conformation of the gyrase B ATP-binding domain. J. Biol. Chem. **277**, 18947–18953 (2002). 11850422 10.1074/jbc.M111740200

[57] K. D. Corbett, J. M. Berger, Structure of the topoisomerase VI-B subunit: Implications for type II topoisomerase mechanism and evolution. Embo J. **22**, 151–163 (2003). 12505993 10.1093/emboj/cdg008 PMC140052

[58] A. Gubaev, D. Klostermeier, DNA-induced narrowing of the gyrase N-gate coordinates T-segment capture and strand passage. Proc. Natl. Acad. Sci. U.S.A. **108**, 14085–14090 (2011). 21817063 10.1073/pnas.1102100108 PMC3161603

[59] T. K. Li, L. F. Liu, Modulation of gyrase-mediated DNA cleavage and cell killing by ATP. Antimicrob. Agents Chemother. **42**, 1022–1027 (1998). 9593120 10.1128/aac.42.5.1022 PMC105738

[60] J. G. Heddle, S. Mitelheiser, A. Maxwell, N. H. Thomson, Nucleotide binding to DNA gyrase causes loss of DNA wrap. J. Mol. Biol. **337**, 597–610 (2004). 15019780 10.1016/j.jmb.2004.01.049

[61] C. L. Baird, T. T. Harkins, S. K. Morris, J. E. Lindsley, Topoisomerase II drives DNA transport by hydrolyzing one ATP. Proc. Natl. Acad. Sci. U.S.A. **96**, 13685–13690 (1999). 10570133 10.1073/pnas.96.24.13685 PMC24125

[62] K. M. Soczek, T. Grant, P. B. Rosenthal, A. Mondragon, CryoEM structures of open dimers of gyrase A in complex with DNA illuminate mechanism of strand passage. Elife **7**, e41215 (2018). 30457554 10.7554/eLife.41215 PMC6286129

[63] J. Papillon , Structural insight into negative DNA supercoiling by DNA gyrase, a bacterial type 2A DNA topoisomerase. Nucleic Acids Res. **41**, 7815–7827 (2013). 23804759 10.1093/nar/gkt560 PMC3763546

[64] A. Gubaev, D. Klostermeier, DNA-induced narrowing of the gyrase N-gate coordinates T-segment capture and strand passage. Proc. Natl. Acad. Sci. U.S.A. **108**, 14085–14090 (2011). 21817063 10.1073/pnas.1102100108 PMC3161603

[65] I. Laponogov , Trapping of the transport-segment DNA by the ATPase domains of a type II topoisomerase. Nat. Commun. **9**, 2579 (2018). 29968711 10.1038/s41467-018-05005-x PMC6030046

[66] B. H. Schmidt, N. Osheroff, J. M. Berger, Structure of a topoisomerase II-DNA-nucleotide complex reveals a new control mechanism for ATPase activity. Nat. Struct. Mol. Biol. **19**, 1147 (2012). 23022727 10.1038/nsmb.2388 PMC3492516

[67] A. Vanden Broeck , Structural basis for allosteric regulation of Human Topoisomerase IIα. Nat. Commun. **12**, 2962 (2021). 34016969 10.1038/s41467-021-23136-6 PMC8137924

[68] S. Hartmann, D. Weidlich, D. Klostermeier, Single-molecule confocal FRET microscopy to dissect conformational changes in the catalytic cycle of DNA topoisomerases. Method Enzymol. **581**, 317–351 (2016). 10.1016/bs.mie.2016.08.013 27793284

[69] N. S. Rovinskiy, A. A. Agbleke, O. N. Chesnokova, N. P. Higgins, Supercoil levels in *E. coli* and *Salmonella* chromosomes are regulated by the C-Terminal 35–38 Amino Acids of GyrA. Microorganisms **7**, 81 (2019). 30875939 10.3390/microorganisms7030081 PMC6463007

[70] F. M. Heydenreich , High-throughput mutagenesis using a two-fragment PCR approach. Sci. Rep-Uk **7**, e3484 (2017). 10.1038/s41598-017-07010-4 PMC5533798 28754896

[71] A. Punjani, J. L. Rubinstein, D. J. Fleet, M. A. Brubaker, cryoSPARC: Algorithms for rapid unsupervised cryo-EM structure determination. Nat. Methods **14**, 290–296 (2017). 28165473 10.1038/nmeth.4169

[72] A. Punjani, H. Zhang, D. J. Fleet, Non-uniform refinement: Adaptive regularization improves single-particle cryo-EM reconstruction. Nat. Methods **17**, 1214–1221 (2020). 33257830 10.1038/s41592-020-00990-8

[73] J. Zivanov, T. Nakane, S. H. W. Scheres, A Bayesian approach to beam-induced motion correction in cryo-EM single-particle analysis. IUCrj **6**, 5–17 (2019). 30713699 10.1107/S205225251801463X PMC6327179

[74] J. Zivanov, T. Nakane, S. H. W. Scheres, Estimation of high-order aberrations and anisotropic magnification from cryo-EM data sets in RELION-3.1. IUCrJ **7**, 253–267 (2020). 10.1107/S2052252520000081 PMC7055373 32148853

[75] M. Wolf, D. J. DeRosier, N. Grigorieff, Ewald sphere correction for single-particle electron microscopy. Ultramicroscopy **106**, 376–382 (2006). 16384646 10.1016/j.ultramic.2005.11.001

[76] T. Bepler , Positive-unlabeled convolutional neural networks for particle picking in cryo-electron micrographs. Nat. Methods **16**, 1153 (2019). 31591578 10.1038/s41592-019-0575-8 PMC6858545

[77] P. Emsley, B. Lohkamp, W. G. Scott, K. Cowtan, Features and development of Coot. Acta Crystallogr. D. Biol. Crystallogr. **66**, 486–501 (2010). 20383002 10.1107/S0907444910007493 PMC2852313

[78] K. Jamali , Automated model building and protein identification in cryo-EM maps. Nature **628**, 450-457 (2024). 38408488 10.1038/s41586-024-07215-4 PMC11006616

[79] T. I. Croll, a physically realistic environment for model building into low-resolution electron-density maps. Acta Crystallogr. D. **74**, 519–530 (2018). 10.1107/S2059798318002425 PMC6096486 29872003

[80] X. Wang, G. Terashi, D. Kihara, CryoREAD: de novo structure modeling for nucleic acids in cryo-EM maps using deep learning. Nat. Methods **20**, 1739–1747 (2023). 37783885 10.1038/s41592-023-02032-5 PMC10841814

[81] D. Liebschner , Macromolecular structure determination using X-rays, neutrons and electrons: Recent developments in Phenix. Acta Crystallogr. D. Struct. Biol. **75**, 861–877 (2019). 31588918 10.1107/S2059798319011471 PMC6778852

[82] E. F. Pettersen , UCSF ChimeraX: Structure visualization for researchers, educators, and developers. Protein Sci. **30**, 70–82 (2021). 32881101 10.1002/pro.3943 PMC7737788

[83] J. A. Ali, A. P. Jackson, A. J. Howells, A. Maxwell, The 43-kilodalton N-Terminal fragment of the DNA gyrase-B protein hydrolyzes ATP and binds coumarin drugs. Biochemistry **32**, 2717–2724 (1993). 8383523 10.1021/bi00061a033

[84] A. Iudin , EMPIAR: The electron microscopy public image archive. Nucleic Acids Res. **51**, D1503–D1511 (2023). 36440762 10.1093/nar/gkac1062 PMC9825465

