# Supplementary material for: Structural basis of chiral wrap and T-segment capture by Escherichia coli DNA gyrase
Source: Proc Natl Acad Sci U S A. 2024 Nov 26;121(49):e2407398121. doi: 10.1073/pnas.2407398121 (PMC11626157; doi:10.1073/pnas.2407398121)
Supplement: Supplementary file 1 — Appendix 01 (PDF) [file pnas.2407398121.sapp.pdf]

Supporting Information for

Structural basis of chiral wrap and T-segment capture by *Escherichia coli* DNA gyrase

Elizabeth Michalczyk[1,2], Zuzanna Pakosz-Stępień[3], Jonathon D. Liston[3], Olivia Gittins[3], Marta Pabis[*2], Jonathan G. Heddle*[2,3] and Dmitry Ghilarov*[4]

[1]Doctoral School of Exact and Natural Sciences, Jagiellonian University, 30-348, Kraków, Poland; [2]Malopolska Centre of Biotechnology, Jagiellonian University, 30-387, Kraków, Poland; [3]Centre for Programmable Biological Matter, School of Biological and Biomedical Sciences, Durham University, DH1 3LE, Durham, UK; [4]Department of Molecular Microbiology, John Innes Centre, NR4 7UH, Norwich, UK.

Correspondence to: Marta Pabis, Jonathan G. Heddle, Dmitry Ghilarov
Email: *dmitry.ghilarov@jic.ac.uk; marta.pabis@uj.edu.pl; jonathan.g.heddle@durham.ac.uk*

This PDF file includes:

Figures S1 to S15
Tables S1 to S2
Legends for Movies S1 to S2
SI References

Other supporting materials for this manuscript include the following:

Movies S1 to S2

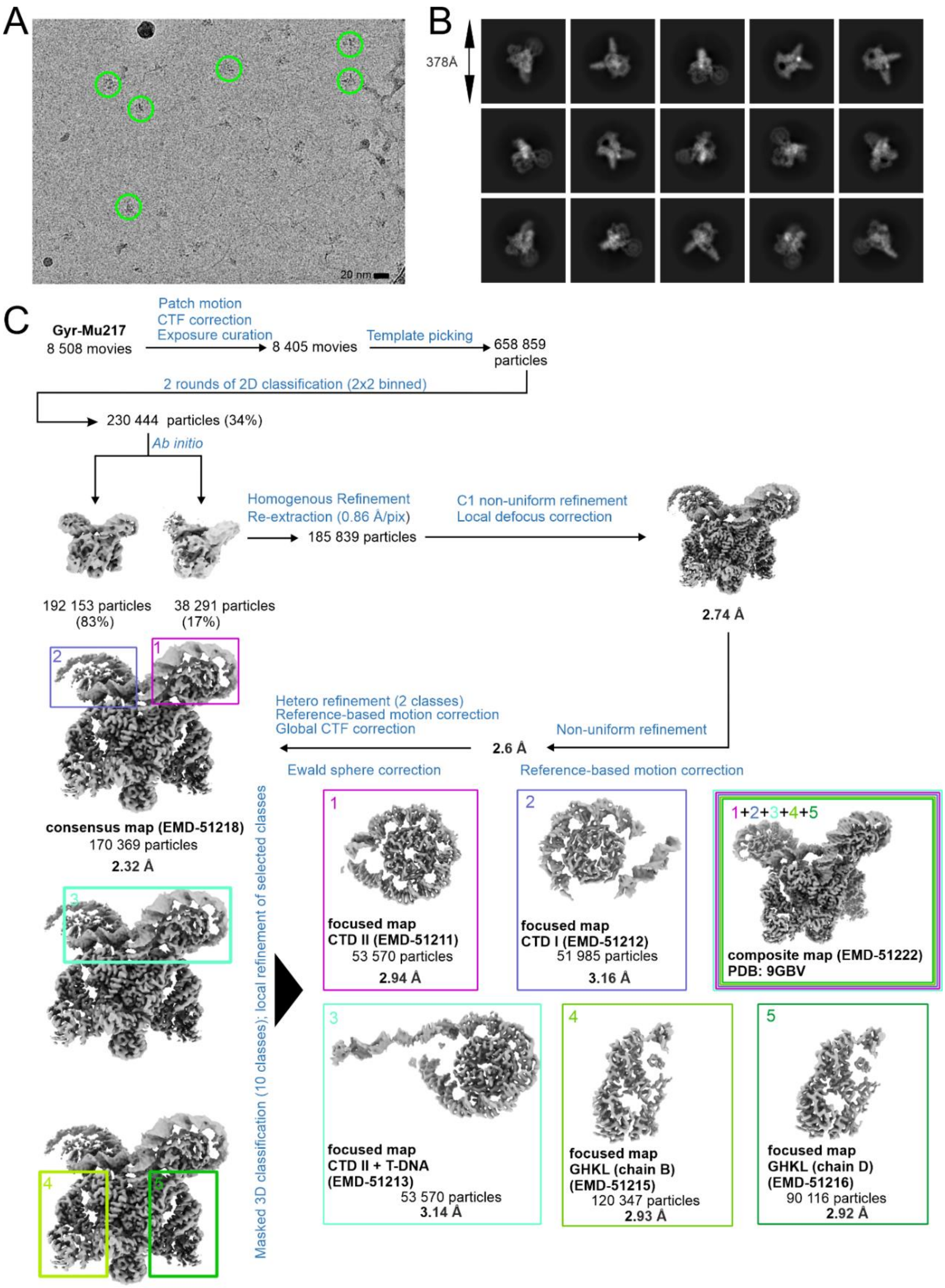

Fig. S1. CryoEM data processing scheme for **Gyr-Mu217**. (**A**) A representative motion-corrected micrograph with gyrase particles encircled. (**B**) A selection of 2D classes, box size in angstroms indicated. (**C**) Processing scheme (see **Methods** for description). All processing was done in cryoSPARC. Colored boxes represent five regions of the consensus map where masking, focused 3D classification and focused refinement were used to improve map quality. All local maps were fitted in the consensus map, and combined to obtain the composite map used for final refinement.

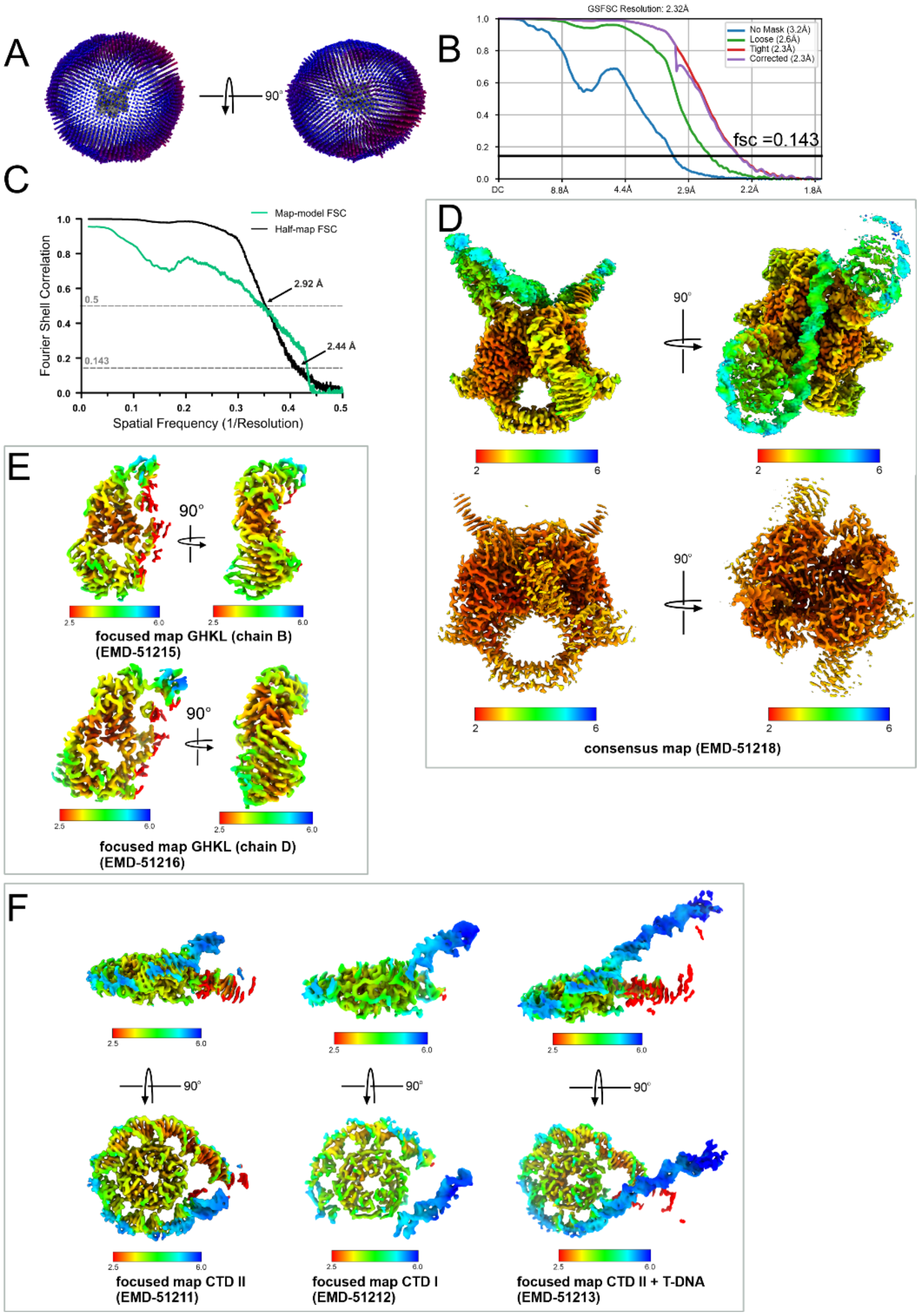

Fig. S2. CryoEM data validation for Gyr-Mu217. (**A**) An angular distribution plot as output by cryoSPARC. (**B**) FSC curve for the final reconstruction as output by cryoSPARC. (**C**) Map-to-model FSC curve (green) compared with half-maps FSC curve (black) as output by Phenix (soft mask based on the atomic model is used). FSC=0.5 (for map-to-model FSC) and FSC=0.143 (for half-map FSC) values are indicated with arrows. (**D**) Local resolution maps for the consensus map contoured at two different levels (σ9 and σ20) illustrate resolution distribution from 2.1 Å next to the DNA, to >5 Å towards the ends of the wrapped DNA molecule and flexible CTD domains. (**E**) Local resolution maps for GHKL domains. (**F**) Local resolution maps for CTDs **I** and **II** and for T-DNA.

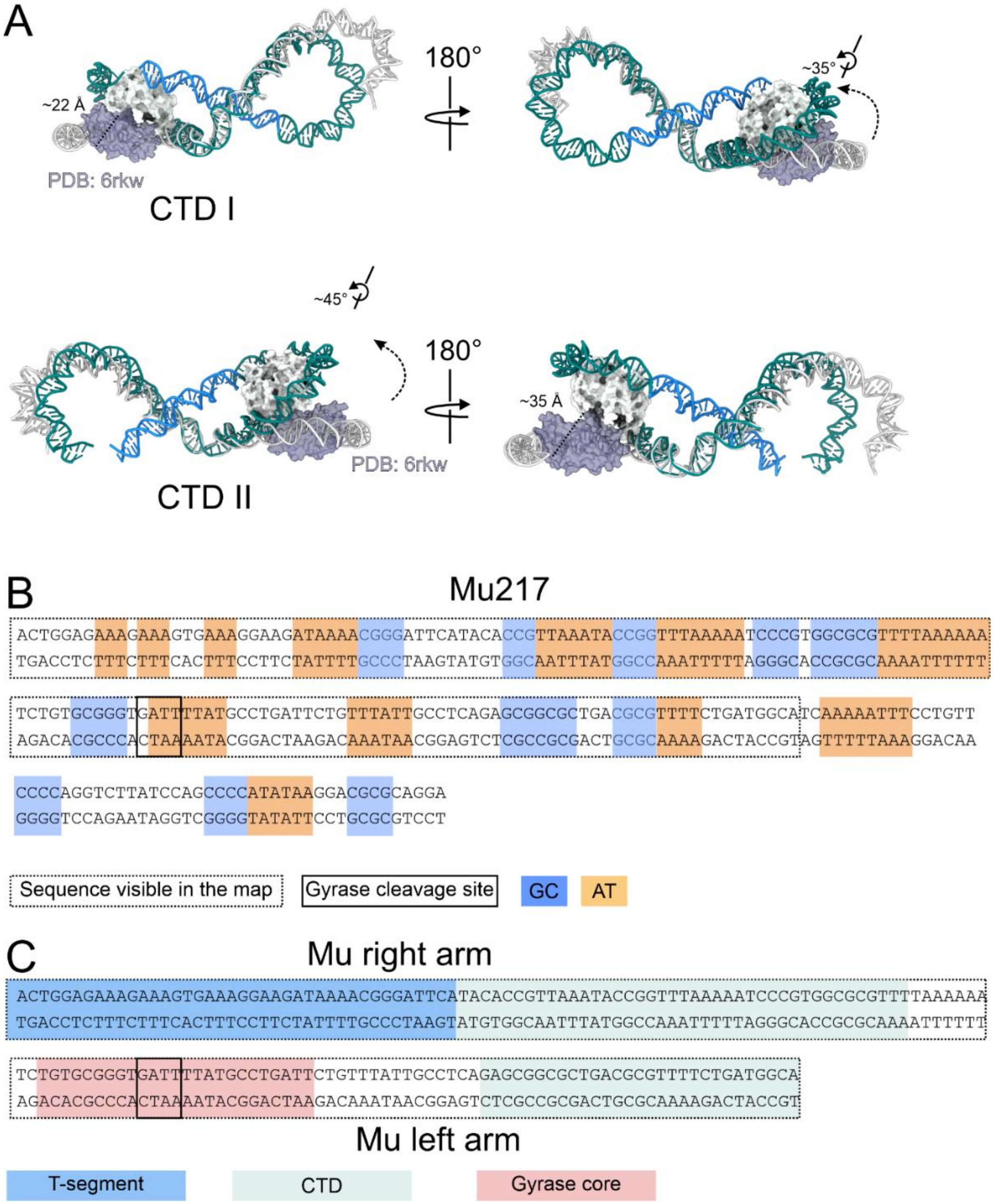

Fig. S3. Conformation of CTDs and Mu217 DNA sequence. (**A**) A comparison of the positions of the CTDs and DNA in the Gepotidacin and AMP-PNP bound structure (PDB: 6RKW) superimposed on the current chirally wrapped structure (PDB: 9GBV). For PDB: 6RKW, DNA (cartoon representation) is colored gray and CTD (surface representation) transparent purple. For the chirally wrapped structure, DNA and CTDs are colored according to **Figure 1** (G-DNA: green, T-DNA: blue, CTD: solid mint white). CTDs are moving upwards with a 35-45° angular rotation and a 20-35 Å shift as measured by the distance between the C-terminus of the CTD. (**B**) Sequence of Mu217 DNA used in this work (1, 2). Boxes show the cleavage site (GATT) and the modeled part of the sequence. GC- and TA-repeats (3 nt and longer) are shown in blue and orange, respectively. (**C**) Sequence of Mu217 DNA with fragments of DNA interacting with the gyrase core, CTDs and presented as the T-segment shown. 'Right arm' and 'left arm' are indicated(3).

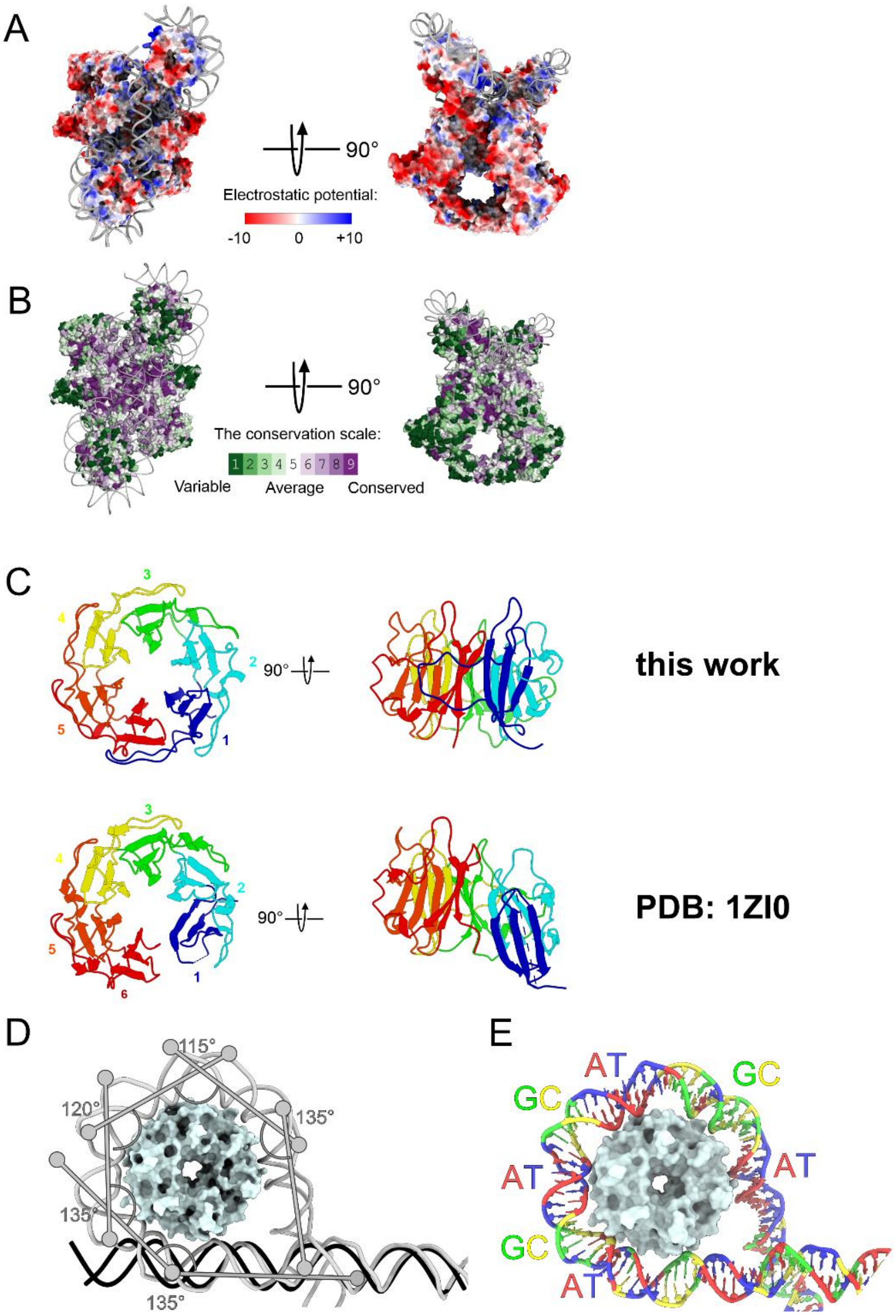

Fig. S4. Gyr-Mu217 surface charge. (**A**) A molecular surface representation of **Gyr-Mu217** model colored by Coulombic potential as calculated by the ChimeraX *coulombic* module using default parameters (in units of kcal/(mol·e). DNA is shown as cartoon grey representation. Blue corresponds to positive charge and red to negative charge. A positively charged guiding pathway for the T-segment is visible. (**B**) An equivalent representation colored by conservation value according to ConSurf server (4). (**C**) A comparison of β-pinwheel domains modeled in this work (**Gyr-Mu217**) and based on the structure of an isolated CTD(PDB: 1ZI0) (5). Individual blades are colored. (**D**) An illustration of DNA bending around the CTD. Five bends introduce a total ~260° angle in DNA. (**E**) Sequence-specificity of DNA wrapping. AT-rich parts of the Mu217 right arm are positioned to form the minor groove facing the protein, while the GC-rich minor grooves face outwards, reminiscent of a nucleosome DNA wrap.

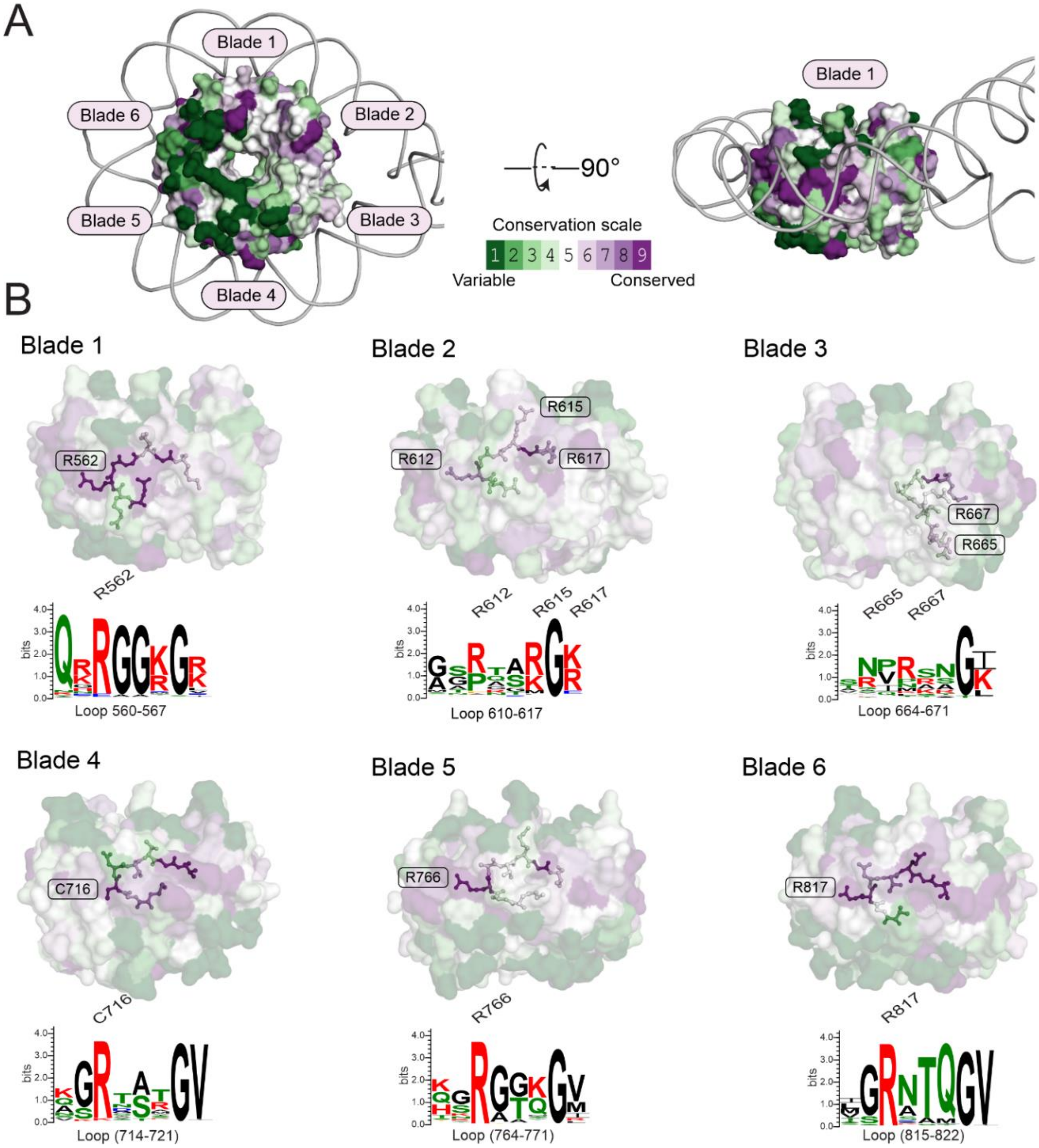

Fig. S5. Conservation analysis of GyrA C-terminal domain. (**A**) GyrA C-terminal domain (modeled based on **Gyr-Mu217**) surface representation colored by conservation according to ConSurf web server (4). Blades are numbered. (**B**) For each blade, a conserved loop interacting with DNA (GyrA-box motif) is shown with residues colored by conservation. Below is the logo representation of the alignment produced by ConSurf generated by WebLogo 3.7.12 (6). The residues mutated in our work (R615, R617, R665, R667) and in a previous work (R612, C716, R766, R817)(7) are indicated. R562 from the GyrA-box in Blade 1(8) was not mutated in isolation but indicated for comparison.

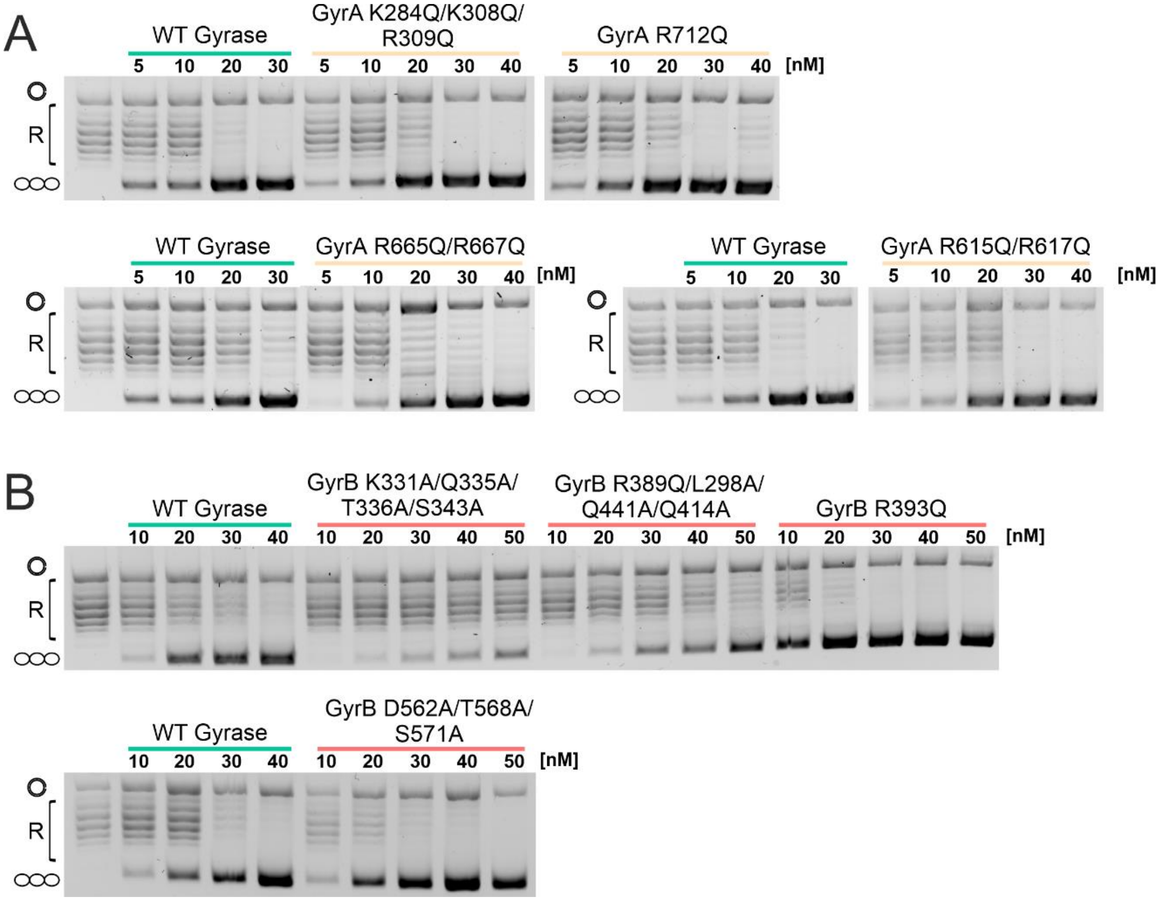

Fig. S6. Supercoiling activity of gyrase variants characterized in this study. (**A**) Plasmid supercoiling assays showing the activity of WT (A_2B_2) gyrase and GyrA variants produced in this work complexed with the WT GyrB subunit. First lane: relaxed pBR322, subsequent lanes: effect of increasing enzyme concentration (as indicated, nM) on relaxed plasmid. Positions of nicked, relaxed and supercoiled DNA are indicated to the left of each gel. Each gel is a representative of at least three biological replicates. (**B**) Plasmid supercoiling assays showing the activity of WT (A_2B_2) gyrase and GyrB variants produced in this work complexed with the WT GyrA subunit. First lane: relaxed pBR322, subsequent lanes: effect of increasing enzyme concentration (as indicated, nM) on relaxed plasmid. Positions of nicked, relaxed and supercoiled DNA are indicated to the left of each gel. Each gel is a representative of at least three biological replicates.

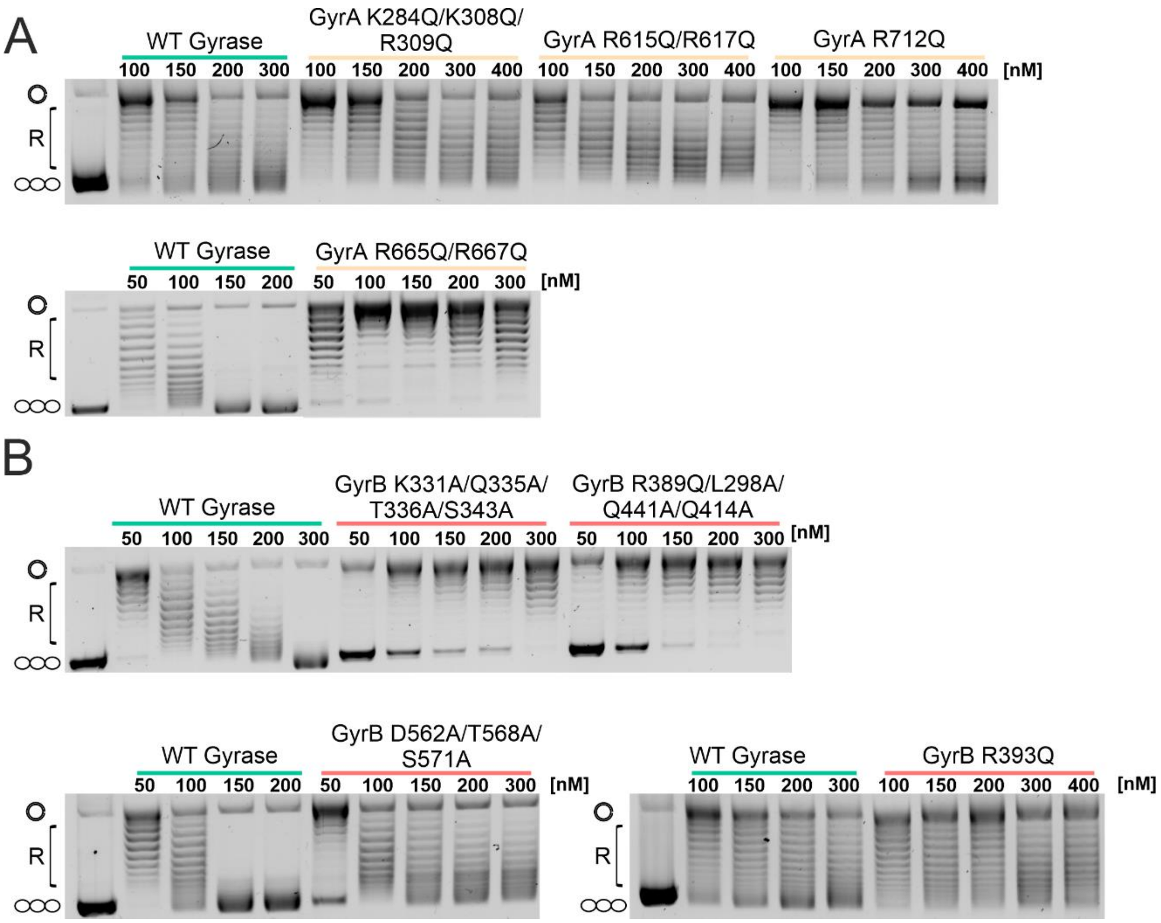

Fig. S7. ATP-independent relaxation activity of gyrase variants characterized in this study. (**A**) Plasmid relaxation assays showing the activity of WT (A_2B_2) gyrase and GyrA variants produced in this work complexed with the WT GyrB subunit. First lane: negatively supercoiled pBR322, subsequent lanes: effect of increasing enzyme concentration (as indicated, nM) on negatively supercoiled plasmid. Positions of nicked, relaxed and supercoiled DNA are indicated to the left of each gel. Supercoiled band visible at very high gyrase concentrations (>150 nM for WT) is a positively supercoiled plasmid. Each gel is a representative of at least three biological replicates. (**B**) Plasmid relaxation assays showing the activity of WT (A_2B_2) gyrase and GyrB variants produced in this work complexed with the WT GyrA subunit. First lane: negatively supercoiled pBR322, subsequent lanes: effect of increasing enzyme concentration (as indicated, nM) on negatively supercoiled plasmid. Positions of nicked, relaxed and supercoiled DNA are indicated to the left of each gel. Each gel is a representative of at least three biological replicates.

A

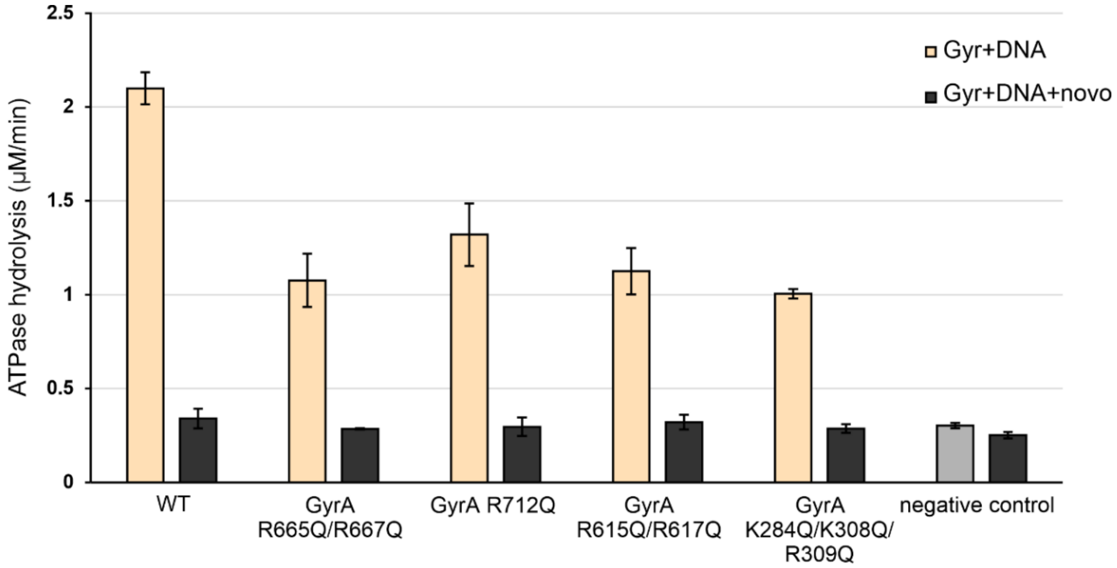

B

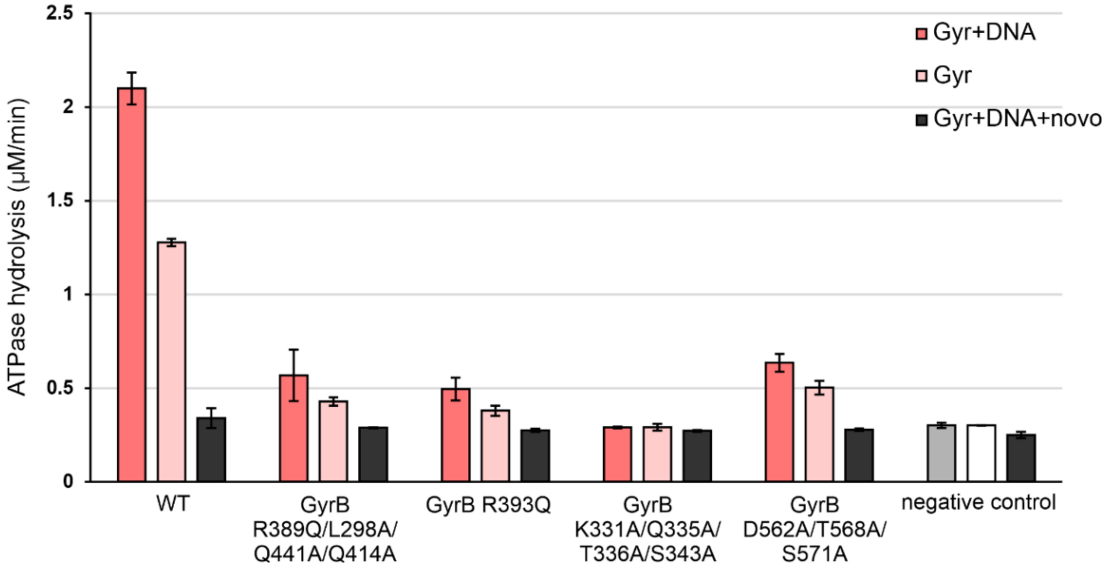

Fig. S8. ATPase activity of gyrase variants characterized in this study. (**A**) DNA-stimulated ATPase activity for 50 nM WT gyrase (A_2B_2) complex mixed with 10.5 nM DNA, or for the respective GyrA variants complexed with the WT GyrB subunit. Novobiocin (novo) was used as a control for background activity. Negative control contained enzyme buffer. Error bars are expressed as the standard deviation of three independent experiments for the rate (mmol/min). (**B**) DNA-independent (pale pink) and DNA-stimulated (pink) ATPase activity for 50 nM WT gyrase (A_2B_2) complex mixed with 10.5 nM DNA, or for the respective GyrB variants complexed with the WT GyrA subunit. Novobiocin (novo) was used as a control for background activity. Negative control contained enzyme buffer with or without DNA. Error bars represent standard deviation of three independent experiments for the rate (mmol/min).

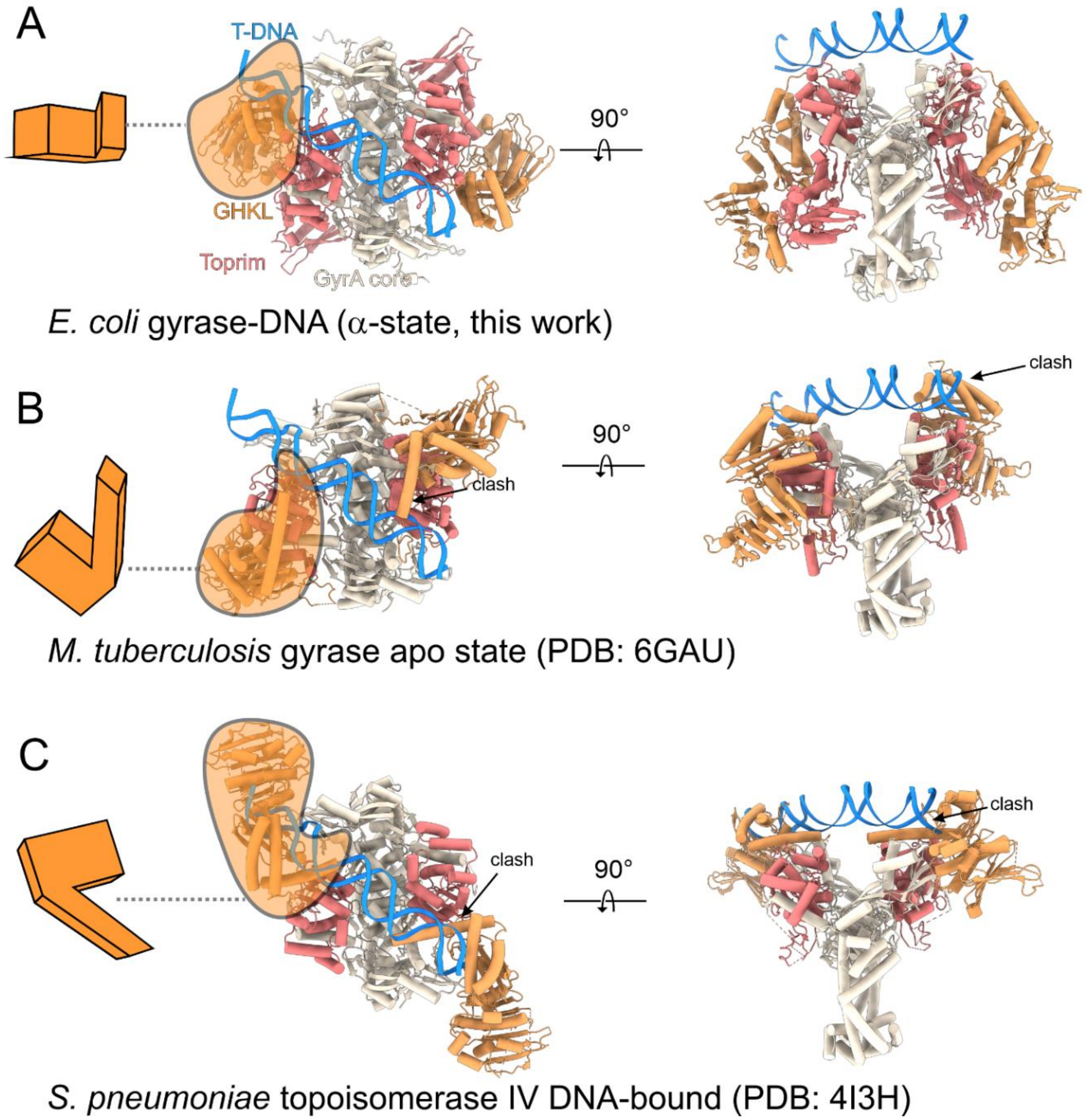

Fig. S9. Comparison of the position of GHKL domains in different type IIA topoisomerase structures. (**A**) **Gyr-Mu217** (current work; PDB: 9GBV). An orange cartoon at the left illustrates the orientation of the GHKL: the 'inner' surface faces the reader. The same coloring scheme is used as in other main figures. (**B**) An 'open-clamp' *M. tuberculosis* gyrase structure (PDB: 6GAU*(9)*) is superimposed based on the GyrA protein, whilst the T-segment orientation is kept intact. The side surface of the GHKL is facing the reader, and clashes with the T-segment. (**C**) An *S. pneumoniae* topoisomerase IV structure (PDB: 4I3H (10)) is superimposed based on the ParC protein, while the position of the T-segment is kept. Once again, the side surface of the GHKL is clashing with the T-segment.

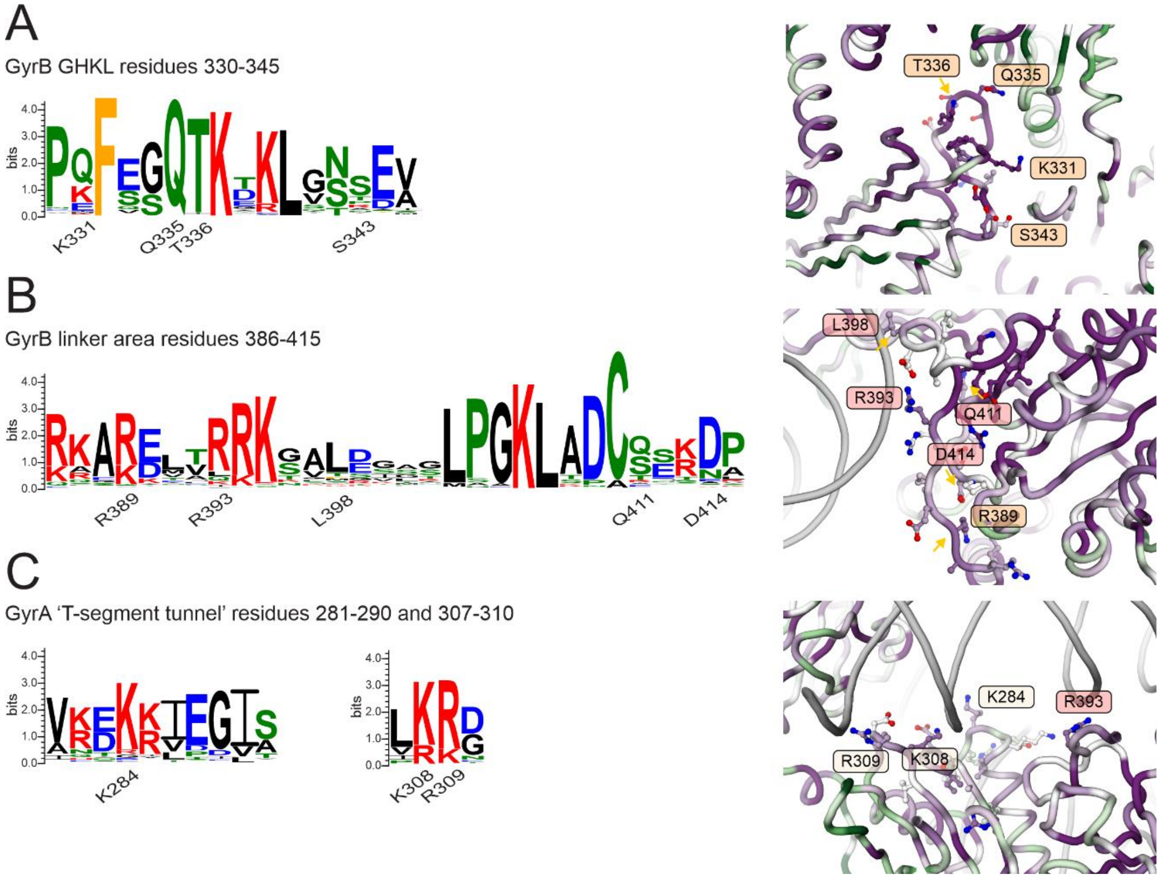

Fig. S10. Conservation analysis of sequences in the GyrA 'T-segment tunnel' and GyrB:GyrB interface. Conservation of residues in GyrB GHKL (**A**), GyrB linker (**B**) and GyrA 'T-segment tunnel' (**C**) shown by the logo representation of the alignment produced by ConSurf (4) generated by WebLogo 3.7.12 (6). Nonpolar aliphatic R groups (G,A,V,L,M,I) are colored black, polar uncharged R groups (S,T,C,P,N,Q) – green, positively charged R groups (K,R,H) - red, negatively charged R groups (D,E) -blue, nonpolar aromatic R groups (F,Y,W) – orange. On the right, cartoon representation (modeled based on **Gyr-Mu217**) colored by conservation according to ConSurf web server. Mutated residues are labeled.

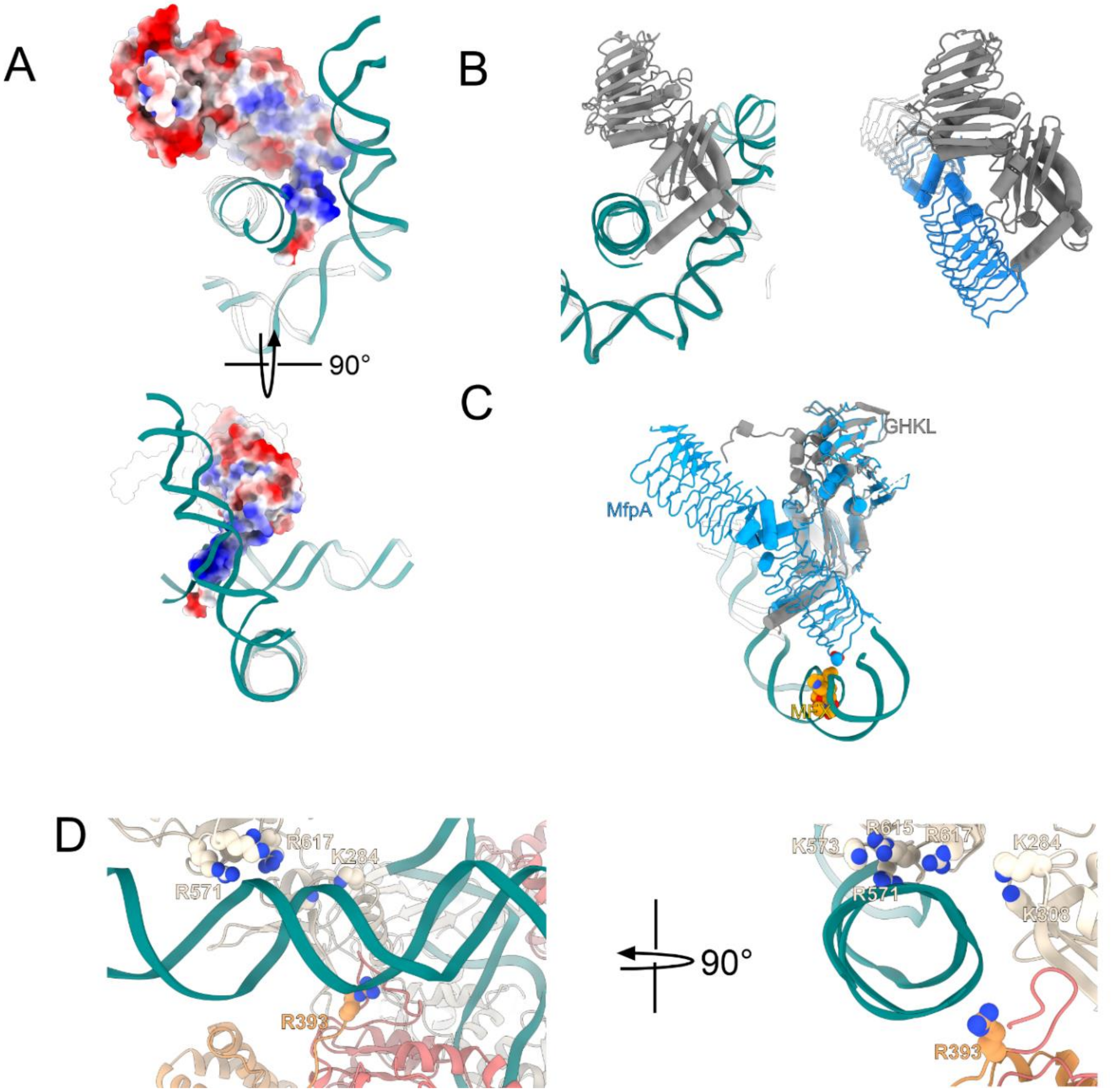

Fig. S11. Comparison of the nucleotide-bound GHKL domain position relative to the T-segment and T-segment mimic, antibiotic resistance protein MfpA. (**A**) A superposition of the GHKL bound to AMP-PNP (only one monomer is shown for clarity; PDB: 6RKW (11)) and the positively supercoiled DNA loop from **Gyr-Mu217** (PDB: 9GBV, current study; colored teal). The GHKL is shown as a Coulomb potential colored surface representation. Two DNA binding surfaces are visible. (**B**) (*left*) A superposition of the GHKL bound to AMP-PNP (PDB: 6RKW (11); GHKL is gray and DNA transparent) and the positively supercoiled DNA loop from **Gyr-Mu217** (PDB: 9GBV, current study; colored teal) and a comparison with (*right*) *Mycobacterium smegmatis* GHKL in complex with MfpA in the same orientation (PDB: 6ZT5 (12); GHKL is gray and MfpA blue). Note the similarity in position of the MfpA molecule and the T-segment. (**C**) A superposition of the MfpA-GyrB47 crystal structure (PDB: 6ZT5 (12); colored blue) and **Gyr-Mu217-MFX** (PDB: 9GGQ, current study). MFX and MfpA D24 are shown as van der Waals spheres. MfpA can reach the MFX molecule, offering a potential explanation for the nucleotide hydrolysis-dependent resistance mechanism. (**D**) An illustration of the role of GyrA Tower and GyrB linker domains in forming a 'tunnel' for the T-segment.

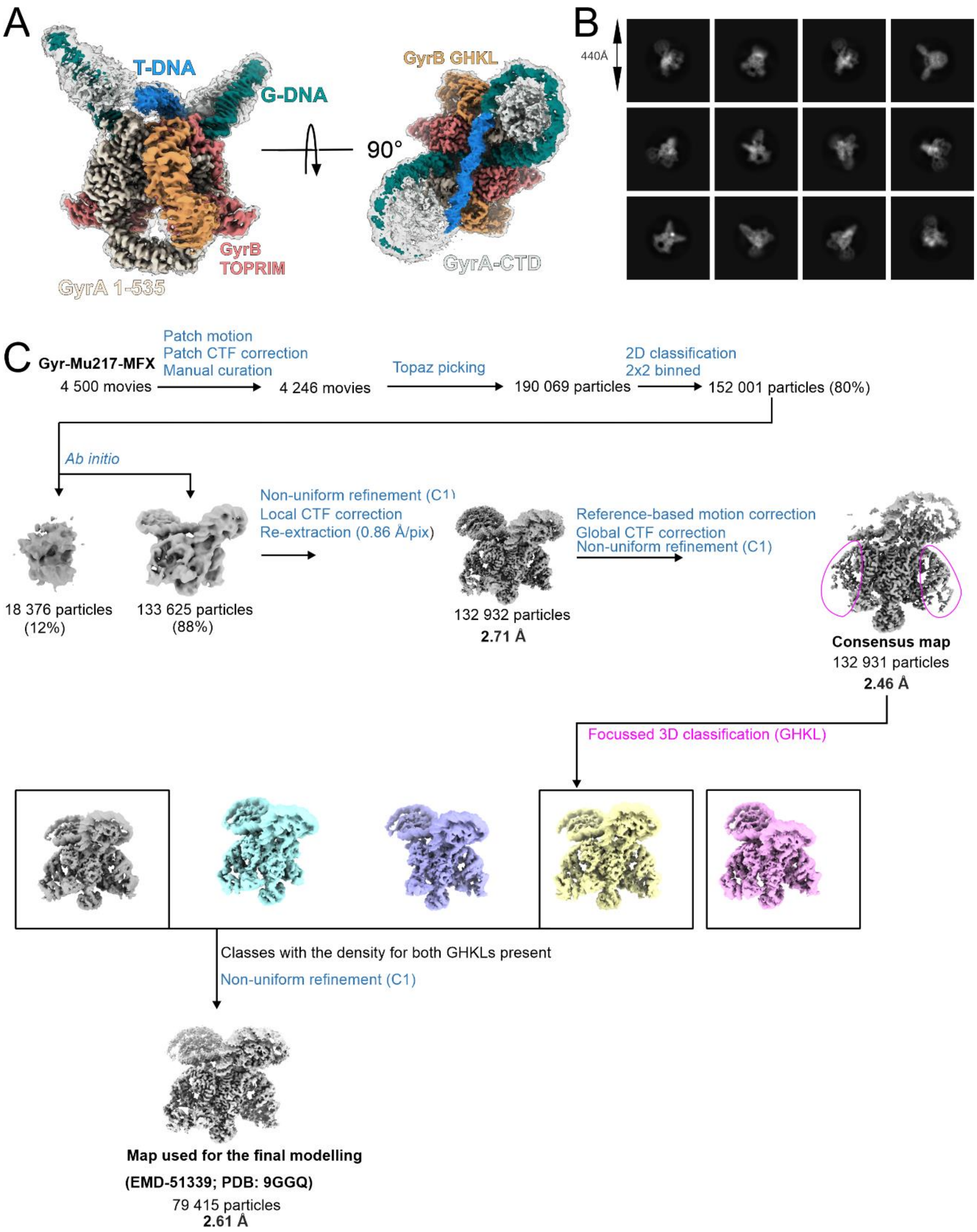

Fig. S12. CryoEM data processing scheme for Gyr-Mu217-MFX. (**A**) Different views of the cryoEM density map for the **Gyr-Mu217-MFX** complex presented at two contour levels (9σ and 5σ). The 9σ map is colored according to the color scheme used elsewhere in the manuscript and 5σ in gray. (**B**) A selection of 2D classes, box size in angstroms indicated. (**C**) Processing scheme (see **Methods** for description).

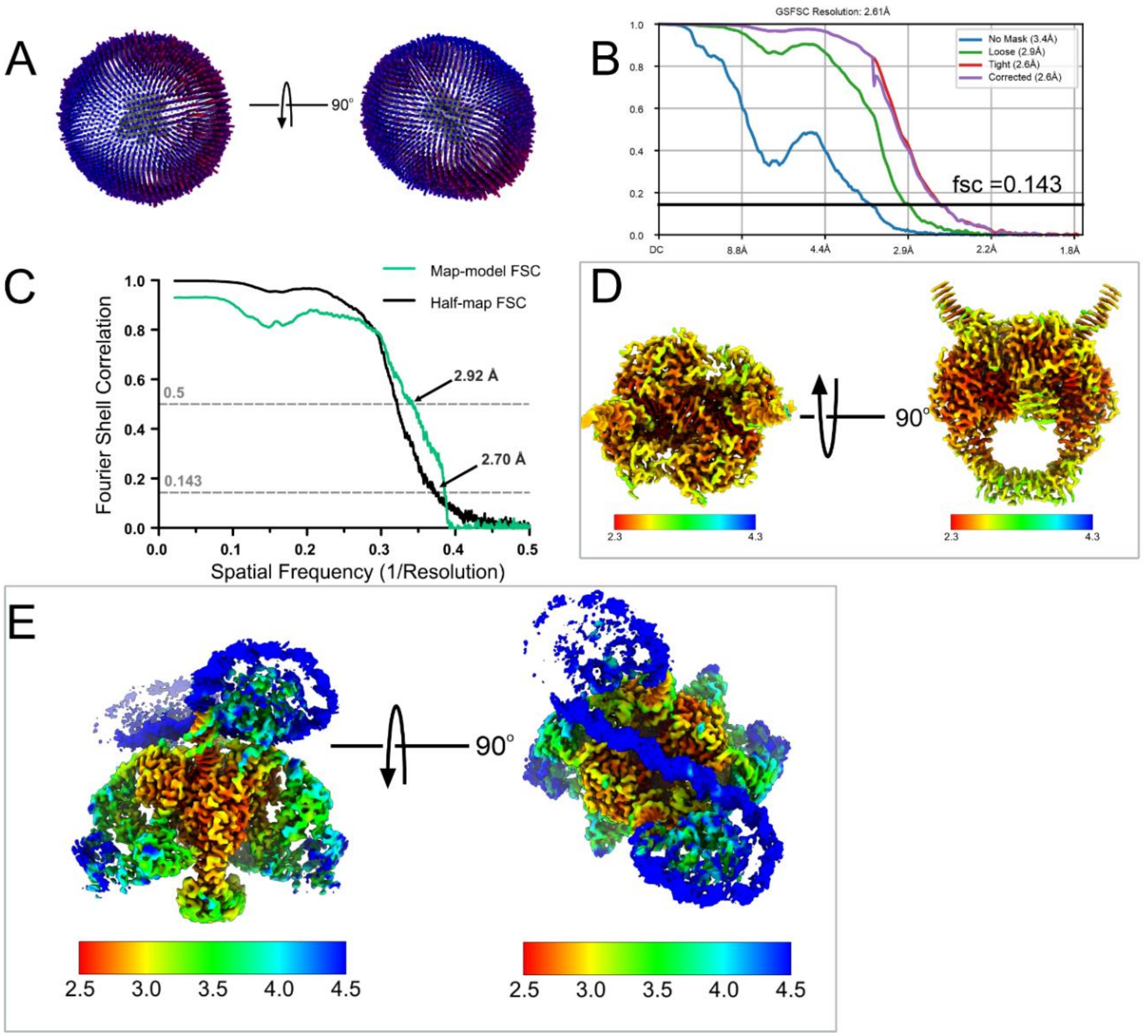

Fig. S13. CryoEM map validation for Gyr-Mu217-MFX. (**A**) Angular distribution diagram as output by cryoSPARC. (**B**) FSC curve for the consensus reconstruction as output by cryoSPARC. (**C**) Map-to-model fit curve. FSC curve (green) compared with half-maps FSC curve (black) as output by Phenix (soft mask based on the atomic model is used). FSC=0.5 (for map-to-model FSC) and FSC=0.143 (for half-map FSC) values are indicated with arrows. (**D**) Local resolution map for the consensus reconstruction (contoured at 11σ). (**E**) Local resolution map for the consensus reconstruction (contoured at 9σ).

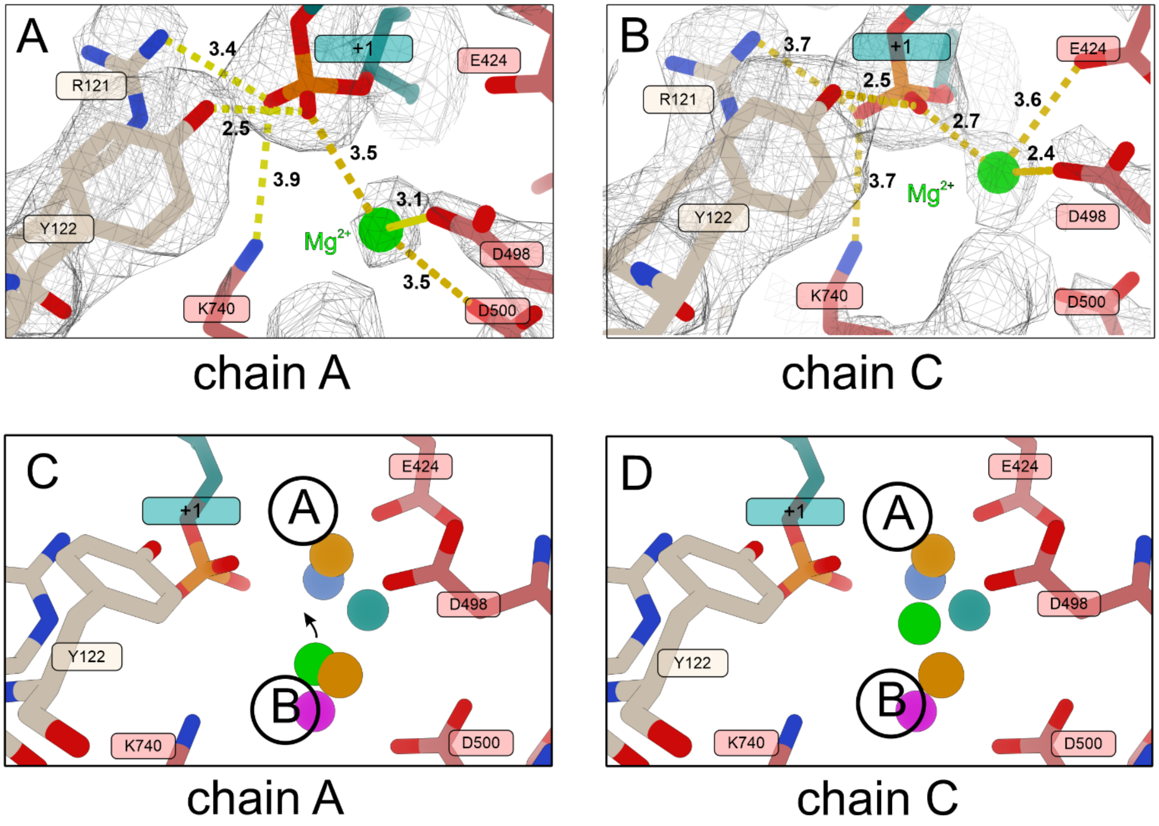

- PDB: 3L4K. Yeast topoisomerase II cleavage complex (cleaved DNA)
- PDB: 9GGQ. This work. *E. coli* DNA gyrase-moxifloxacin complex (cleaved DNA)
- PDB: 3KSA. Topoisomerase IV with cleaved DNA
- PDB: 9GBV. This work. *E. coli* DNA gyrase in a pre-cleavage state
- PDB: 8QQI. *E. coli* DNA gyrase in complex with LEI-800 (intact DNA)

Fig. S14. Comparison of metal ion position in Gyr-Mu217 and other type II topoisomerase structures. Chain A and Chain C in **Gyr-Mu217** are visualised separately to illustrate the subtle differences between the chains. (**A**) Chain A in **Gyr-Mu217**. Catalytic tyrosine, scissile phosphate and Toprim catalytic triad are shown as sticks. Mg^{2+} ion is shown as a lime sphere. Distances in Å are indicated. Density is shown contoured at 15σ. This panel is a copy of panel **E** from **Figure 3** and is reproduced here for reader's convenience. (**B**) Chain C in **Gyr-Mu217**. Note the difference in position of the metal ion which is moving close to the scissile phosphate and is now coordinated by E424 and D498 instead of D500 and D498. (**C**,**D**) Comparison of (**A**,**B**) with other type II topoisomerases: atomic models are superimposed based on the Toprim domain using ChimeraX matchmaker tool. A- and B-type coordination is shown; in structures with uncleaved DNA, metal ions cluster around the A site while in structures with cleaved DNA, they cluster around B site. **Gyr-Mu217** (chain C) places the metal closer to the middle position which is similar to what was reported for the drug-free structure of topoisomerase IV with cleaved and resealed DNA.

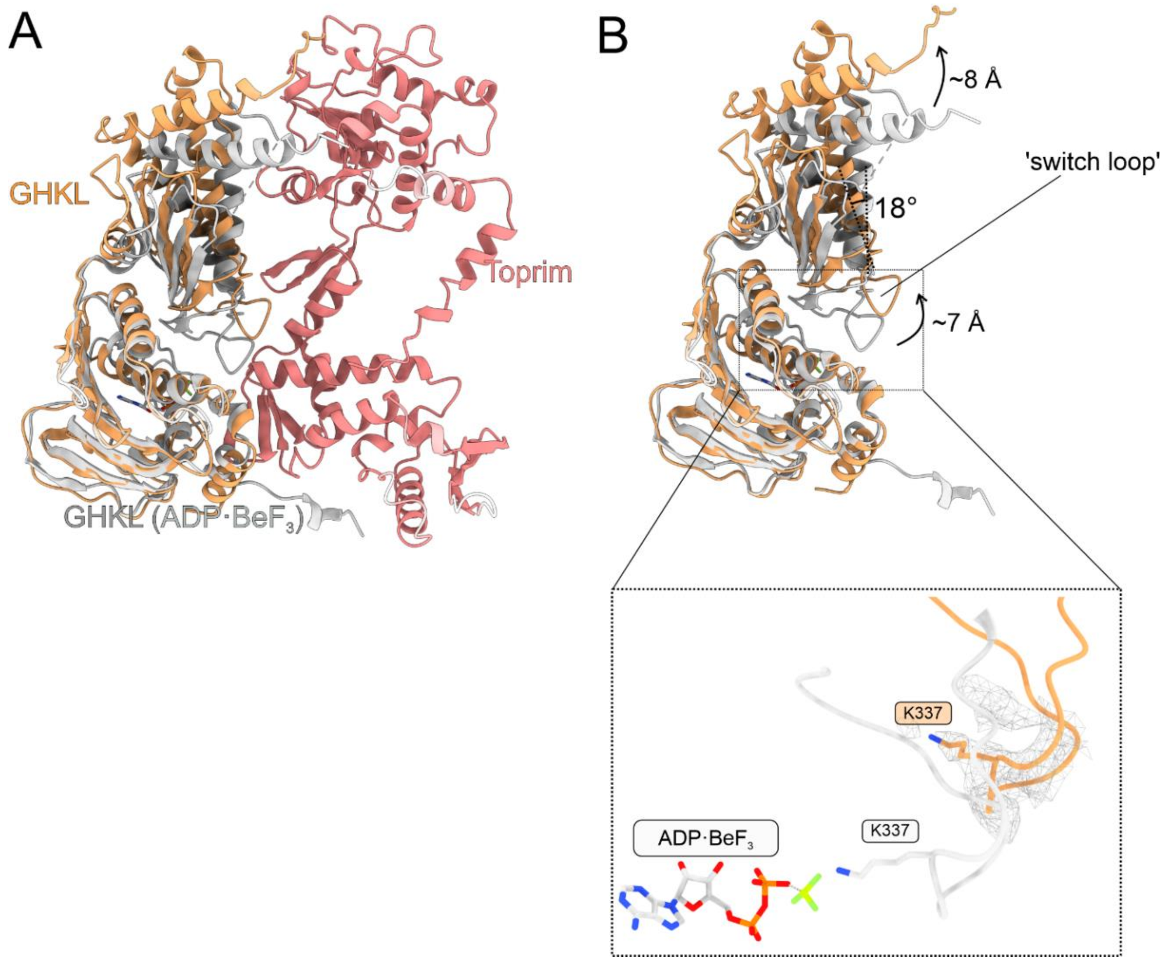

Fig. S15. 'Switch loop' conformation in nucleotide-free structures. (**A**) A superposition of the ATPase domain (GHKL-transducer) in ADP:BeF_3-bound structure of an isolated domain (PDB: 4PU9 (13); white cartoon representation) and the conformation of the same domain in the chirally wrapped (α-state) holocomplex (**Gyr-Mu217**; PDB: 9GBV). The GyrA subunits, opposite GyrB subunit and DNA are not shown. (**B**) The same as A, but with the Toprim removed for clarity, while movements of the transducer helices are highlighted by arrows. Inset shows the 'switch loop' in isolation. In the ADP:BeF_3 structure (PDB: 4PU9), K337 is interacting with BeF_3. Orange cartoon representation shows the equivalent 'switch loop' in the **Gyr-Mu217** structure (PDB: 9GBV). Density (gray mesh) around the loop is shown, supporting modeled lysine orientation.

Table S1. CryoEM data collection and refinement statistics

	**EcGyr-Mu217**	**EcGyr-Mu217-MFX**
PDB code	9GBV	9GGQ
Main EMDB code	EMD-51222 (composite map)	EMD-51339 (consensus map)
**Data collection and processing**		
Microscope	ThermoFisher Krios G3i	ThermoFisher Krios G3i
Magnification	105,000×	105,000×
Voltage (kV)	300	300
Electron dose (e^-/Å^2)	41.84	40.68
Detector	Gatan K3	Gatan K3
Defocus range (-µm)	2.1-0.9	2.1-0.9
Pixel size (Å)	0.86	0.86
Symmetry imposed	C1	C1
Micrographs (no.)	8 508	4 500
Initial particle images (no.)	658 859	190 069
Final particle images (no.)	170 369	79 415
Global map resolution (Å)	2.3 (consensus map)<br>2.9-3.2 (focused maps)	2.6
FSC threshold	0.143	0.143
**Refinement**		
Model resolution (Å)	2.9	2.9
FSC threshold	0.5	0.5
Map sharpening *B* factor (Å^2)	No sharpening performed	No sharpening performed
Model composition<br>Non-hydrogen atoms<br>Protein residues<br>Nucleotides<br>Ligands	 31890<br>3236<br>318<br>2	 24628<br>2905<br>84<br>6
Mean B factors (Å^2)<br>Protein<br>Nucleotide<br>Ligands	 89.31<br>172.64<br>72.25	 141.85<br>78.61<br>71.03
R.m.s. deviations<br>Bond lengths (Å)<br>Bond angles (°)	 0.002<br>0.439	 0.003<br>0.474
**Validation**		
MolProbity score	1.31	1.39
Clashscore	3.96	3.87
Ramachandran plot Favored (%)<br>Allowed (%)<br>Disallowed (%)	97.36<br>2.64<br>0.00	96.61<br>3.39<br>0.00

.

Table S2. Properties of gyrase variants characterized in this work.

Mutations	Rationale	Supercoiling	Relaxation	ATPase activity (% from WT)
GyrA K284Q, K308Q, R309Q	T-segment interaction	Inhibited	Not affected	47.9 ± 1.2
GyrA R615Q, R617Q	CTD wrap (end of wrap)	Inhibited	Not affected	48.7 ± 5.9
GyrA R665Q, R667Q	CTD wrap (beginning of wrap)	Inhibited	Increased	51.3 ± 6.7
GyrA R712Q	CTD wrap (beginning of wrap)	Not affected	Not affected	62.9 ± 8.0
GyrB K331A, Q335A, T336A,S343A	GyrB interface (GHKL)	Inhibited	Inhibited	13.9 ± 0.2
GyrB D562A T568A S571A	GyrB interface (Toprim insert)	Not affected	Inhibited	30.3 ± 2.3
GyrB R389Q, L398A, Q411A, Q414A	GyrB linker	Inhibited	Inhibited	27.1 ± 6.5
GyrB R393Q	T-segment interaction	Increased	Not affected	23.6 ± 2.9

Movie S1 (separate file). Overall structure of Gyr-Mu217. The movie starts by showing the composite cryoEM map for **Gyr-Mu217** (EMD-51222) colored according to the protein domains as in **Figure 1**. The map transitions into a cartoon atomic model (PDB: 9GBV). The protein part of the model fades to show the chiral DNA loop in isolation with T-DNA colored in blue and G-DNA in green.

Movie S2 (separate file). Comparison of PDB: 6RKW and Gyr-Mu217. The movie starts by showing a chirally wrapped DNA loop from PDB: 9GBV (α-state) with G-DNA colored green and T-DNA blue. The DNA fragment from PDB: 6RKW (Ω state) is superimposed in light gray. The dimerised AMP-PNP bound GyrB subunits from the same model (colored pink and gray) are shown to illustrate the clash with the position of T-DNA. Finally, positions of the single GyrB subunit from the two models are compared, PDB: 6RKW colored gray and **Gyr-Mu217** colored pink and orange according to the color scheme in **Figure 1** and throughout the manuscript. The large shift and rotation of the GHKL domain is visible, compared to the almost identical position of the Toprim insertion domain.

SI References

1. K. E. Scheirer, N. P. Higgins, The DNA cleavage reaction of DNA gyrase. Comparison of stable ternary complexes formed with enoxacin and CcdB protein. *J Biol Chem* **272**, 27202-27209 (1997).

2. D. Sutormin, N. Rubanova, M. Logacheva, D. Ghilarov, K. Severinov, Single-nucleotide-resolution mapping of DNA gyrase cleavage sites across the Escherichia coli genome. *Nucleic Acids Res* **47**, 1373-1388 (2019).

3. M. Oram, A. A. Travers, A. J. Howells, A. Maxwell, M. L. Pato, Dissection of the bacteriophage Mu strong gyrase site (SGS): significance of the SGS right arm in Mu biology and DNA gyrase mechanism. *J Bacteriol* **188**, 619-632 (2006).

4. B. Yariv *et al.*, Using evolutionary data to make sense of macromolecules with a "face-lifted" ConSurf. *Protein Science* **32** (2023).

5. A. J. Ruthenburg, D. M. Graybosch, J. C. Huetsch, G. L. Verdine, A superhelical spiral in the DNA gyrase A C-terminal domain imparts unidirectional supercoiling bias. *Journal of Biological Chemistry* **280**, 26177-26184 (2005).

6. G. E. Crooks, G. Hon, J. M. Chandonia, S. E. Brenner, WebLogo: A sequence logo generator. *Genome Res* **14**, 1188-1190 (2004).

7. M. J. Hobson, Z. Bryant, J. M. Berger, Modulated control of DNA supercoiling balance by the DNA-wrapping domain of bacterial gyrase. *Nucleic Acids Research* **48**, 2035-2049 (2020).

8. V. M. Kramlinger, H. Hiasa, The "GyrA-box" is required for the ability of DNA gyrase to wrap DNA and catalyze the supercoiling reaction. *Journal of Biological Chemistry* **281**, 3738-3742 (2006).

9. S. Petrella *et al.*, Overall Structures of DNA Gyrase Reveal the Role of a GyrB-Specific Insert in ATPase Activity. *Structure* **27**, 579-+ (2019).

10. I. Laponogov *et al.*, Structure of an 'open' clamp type II topoisomerase-DNA complex provides a mechanism for DNA capture and transport. *Nucleic Acids Research* **41**, 9911-9923 (2013).

11. A. Vanden Broeck, C. Lotz, J. Ortiz, V. Lamour, Cryo-EM structure of the complete DNA gyrase nucleoprotein complex. *Nature Communications* **10** (2019).

12. L. P. Feng *et al.*, The pentapeptide-repeat protein, MfpA, interacts with mycobacterial DNA gyrase as a DNA T-segment mimic. *P Natl Acad Sci USA* **118** (2021).

13. F. V. Stanger, C. Dehio, T. Schirmer, Structure of the N-Terminal Gyrase B Fragment in Complex with ADP•P Reveals Rigid-Body Motion Induced by ATP Hydrolysis. *Plos One* **9** (2014).
